# Iron Oxyhydroxide-Covalent Organic Framework Nanocomposite for Efficient As(III) Removal in Water

**DOI:** 10.1021/acsami.2c14744

**Published:** 2022-10-25

**Authors:** Ana Guillem-Navajas, Jesús Á. Martín-Illán, Elena Salagre, Enrique G. Michel, David Rodriguez-San-Miguel, Félix Zamora

**Affiliations:** † Departamento de Química Inorgánica, Facultad de Ciencias, Institute for Advanced Research in Chemical Sciences (IAdChem) and Condensed Matter Physics Institute (IFIMAC), Universidad Autónoma de Madrid, Madrid 28049, Spain; ‡ Departamento de Física de la Materia Condensada, Universidad Autónoma de Madrid, Madrid 28048, Spain; § Condensed Matter Physics Center (IFIMAC), Facultad de Ciencias, Universidad Autónoma de Madrid, Madrid 28048, Spain

**Keywords:** COF, arsenic capture, water remediation, nanocomposite, iron oxyhydroxide nanorods

## Abstract

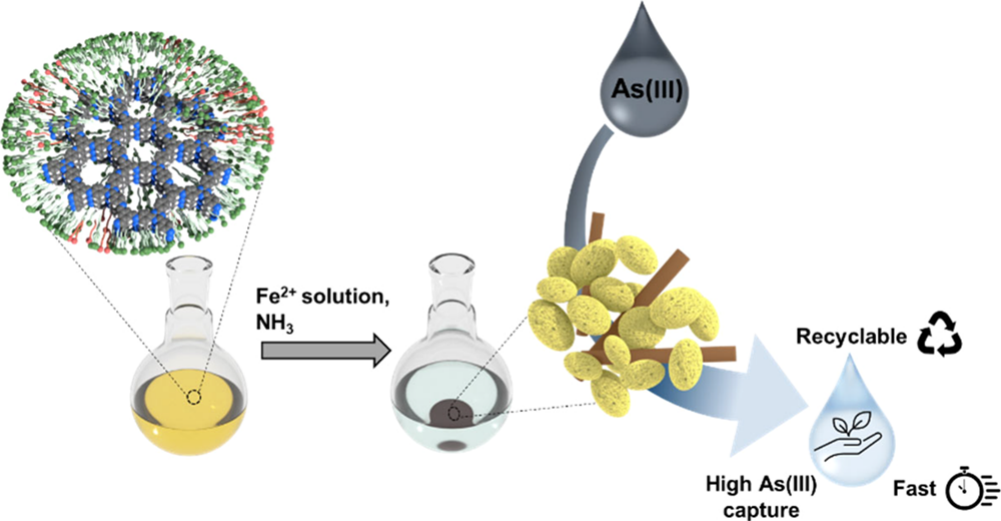

The presence of heavy metal ions in water is an environmental issue derived mainly from industrial and mineral contamination. Metal ions such as Cd(II), Pb(II), Hg(II), or As(III) are a significant health concern worldwide because of their high toxicity, mobility, and persistence. Covalent organic frameworks (COFs) are an emerging class of crystalline organic porous materials that exhibit very interesting properties such as chemical stability, tailored design, and low density. COFs also allow the formation of composites with remarkable features because of the synergistic combination effect of their components. These characteristics make them suitable for various applications, among which water remediation is highly relevant. Herein, we present a novel nanocomposite of iron oxyhydroxide@COF (FeOOH@Tz-COF) in which lepidocrocite (γ-FeOOH) nanorods are embedded in between the COF nanoparticles favoring As(III) remediation in water. The results show a remarkable 98.4% As(III) uptake capacity in a few minutes and impressive removal efficiency in a wide pH range (pH 5–11). The chemical stability of the material in the working pH range and the capability of capturing other toxic heavy metals such as Pb(II) and Hg(II) without interference confirm the potential of FeOOH@Tz-COF as an effective adsorbent for water remediation even under harsh conditions.

## Introduction

Water contamination is currently one of the world’s leading causes of death.^1^ The increase in energy production and the exponential necessity of heavy metal use in industrial processes have caused a rise in human exposure to toxic elements in the last decades. Transition metals such as cadmium, chromium, lead, arsenic, and mercury are among the most concerning ones because they play no role in human homeostasis, induce multiple organ damage, cause birth defects, and are classified as carcinogens.^2^ To maintain environmental and human wellbeing, we must find new solutions for the cheap and energy-efficient remediation of trace contaminants from water.^3^ Among these ground-water pollutants, inorganic arsenic is one of the most significant and problematic because of its high toxicity and mobility over a wide range of conditions. It is estimated that at least 140 million people are being exposed to this chemical and at risk of suffering from skin, lung, or bladder cancer in the future.^4^ Commercial heavy metal remediation methods, such as chemical precipitation, sorbents, and membranes, have many drawbacks: high economic and energetic cost, low removal efficiency, difficult regeneration and/or fouling, and the production of large quantities of chemical sludge. In this context, adsorption is the most promising one because of the low costs and easy implementation.^5^

In the case of As(III) remediation from contaminated water, adsorption is the most extended method, natural amorphous iron oxides^6^ and amorphous carbon^7^ being the traditional adsorbents used in this matter. However, their efficiency and effectiveness are still very poor. Synthetic iron nanoparticles have been produced to enhance arsenic uptake by increasing their surface area,^8^ but they are difficult to recover and regenerate once used, and their selectivity is low because of their high reactivity.^9^ In this context, much effort is being put into the design of new selective and efficient materials to improve the removal of arsenic from contaminated water, such as MOFs,^10,11^ zeolites,^12^ magnesium oxide nanoflakes,^13,14^ polymers,^15^ and covalent organic frameworks (COFs).^16^

COFs are a novel type of crystalline organic porous material in which reversible covalent bonds link organic building blocks.^17^ These materials are characterized by their permanent porosity, high specific surface area, and chemical stability.^18^ These properties make them suitable for many applications such as gas separation, gas storage, energy storage, catalysis, and chemical sensing.^19,20^ In addition, they have been recently used for water treatment as their low density and tunability make them excellent candidates for contaminant removal.^21−23^

COFs also offer the possibility of producing composites by introducing other compounds into their porous structure to incorporate new properties. Their porosity allows the guest material to be accessible while preventing it from aggregating and deactivating. Thus, many functional materials such as metal, metal oxide, or silica nanoparticles have been successfully used to form COF-based composites with remarkable features.^24^

One of the main hurdles in COF composite production is the control of the morphology and particle size. In this regard, a novel method for the production of stable aqueous colloidal COF nanoparticles based on the use of micelles as nanoreactors was recently reported by our group, which offers new possibilities in COF composite synthesis.^25^

Herein, we have successfully obtained an FeOOH@Tz-COF nanocomposite capable of removing As(III) from water very efficiently, overcoming the typical limitations of conventional iron oxides. This material is obtained following an easy two-step synthesis that uses the COF nanoparticles as a template for forming lepidocrocite (γ-FeOOH) nanorods, a structure that presents many active sites for As(III) uptake. The large number of nanorods, their homogeneous distribution, and their exceptionally small size lead to one of the highest experimental uptakes achieved so far at pH = 7 for arsenic adsorbent materials.

## Results and Discussion

**Figure 1:**
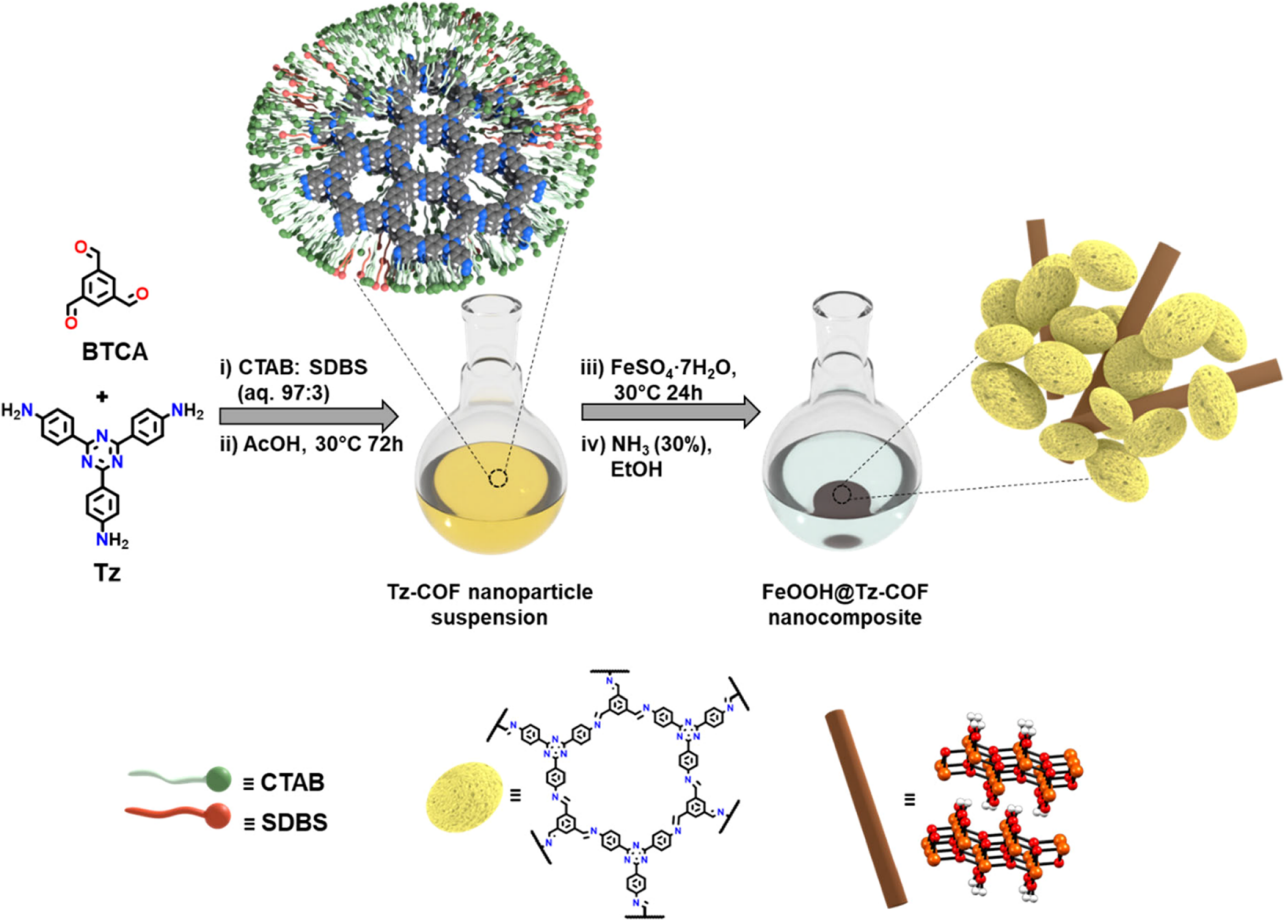
Schematic representation of FeOOH@Tz-COF nanocomposite synthesis. (i) Solubilization of BTCA and Tz in the catanionic micellar system. (ii) Tz-COF nanoparticle suspension formation. (iii) Incorporation of an iron sulfate solution into the suspension. (iv) Flocculation of the nanoparticles and formation of the nanocomposite.

The synthesis of the FeOOH@Tz-COF nanocomposite was achieved in two steps (Figure 1). First, a nanoparticle suspension of Tz-COF was prepared following the procedure for aqueous colloidal suspensions reported by us with slight modifications.^25^ The catanionic micellar system used for this synthesis was a mixture of hexadecyltrimethylammonium bromide (CTAB) and sodium dodecylbenzenesulfonate (SDBS) surfactants in a 97:3 ratio. These conditions allow the complete solubilization of 1,3,5-triformylbenzene (BTCA) and 2,4,6-tris(4-aminophenyl)-1,3,5-triazine (Tz). The poor solubility of Tz was addressed by changing its concentration and the anionic surfactant reported in the previous work (see the Experimental Section). After mixing the two solutions and adding acetic acid (AcOH), the resulting mixture turned yellow and was kept at 30 °C for 3 days. The Willis–Tyndall scattering behavior confirmed the formation of the colloidal suspension (Figure S1, SI), and dynamic light scattering (DLS) measurements demonstrated that the nanoparticle size distribution was monodisperse and centered around 20 nm (Figure S2a, SI).

Then, an aqueous iron(II) sulfate solution (FeSO_4_·7H_2_O, 0.036 mol L^–1^) was added to the Tz-COF suspension and kept undisturbed for 24 h. DLS results showed a nanoparticle size distribution centered at 20 nm, so no aggregation was found after the modification (Figure S2b, SI). Finally, the material was isolated upon adding an ammonia solution (30%) up to pH = 7 and ethanol (EtOH). After flocculation, the color of the suspension changed from yellow to brown because of the formation and incorporation of iron oxyhydroxide into the Tz-COF structure. The resulting brown solid hereafter termed FeOOH@Tz-COF was centrifuged and washed several times with EtOH and activated by supercritical CO_2_ drying (scCO_2_). A control sample of Tz-COF suspension without modification with iron(II) sulfate was also prepared and isolated following this method leading to a yellow powder called Tz-COF(s) (see the Experimental Section).

Both Tz-COF(s) and FeOOH@Tz-COF were characterized by Fourier transform infrared spectroscopy (FT-IR), ^13^C CP-MAS solid-state nuclear magnetic resonance (NMR), thermogravimetric analysis (TGA), and elemental analysis. FT-IR spectra corroborated the presence in both materials of the C=N stretching band associated with the imine bond (1623 cm^–1^) and the aromatic triazine C=N stretching band (1601 cm^–1^) characteristic of Tz-COF. The incorporation of the oxyhydroxide did not seem to affect the COF structure as no significant changes can be observed (Figures S3 and S4, SI). As for the solid-state ^13^C CP-MAS NMR spectra, Tz-COF(s) and FeOOH@Tz-COF showed a signal at 155 ppm associated with the imine carbon atom and a signal at 168.8 ppm that can be assigned to the carbon atom of the triazine ring of Tz (Figure S5, Table S1, SI). No changes were observed after the modification, confirming that the iron oxyhydroxide incorporated did not interact with the imine bonds in the COF structure. The TGA results of both materials in N_2_ atmosphere demonstrated their thermal stability up to 500 °C (Figures S6 and S7, SI).

**Figure 2:**
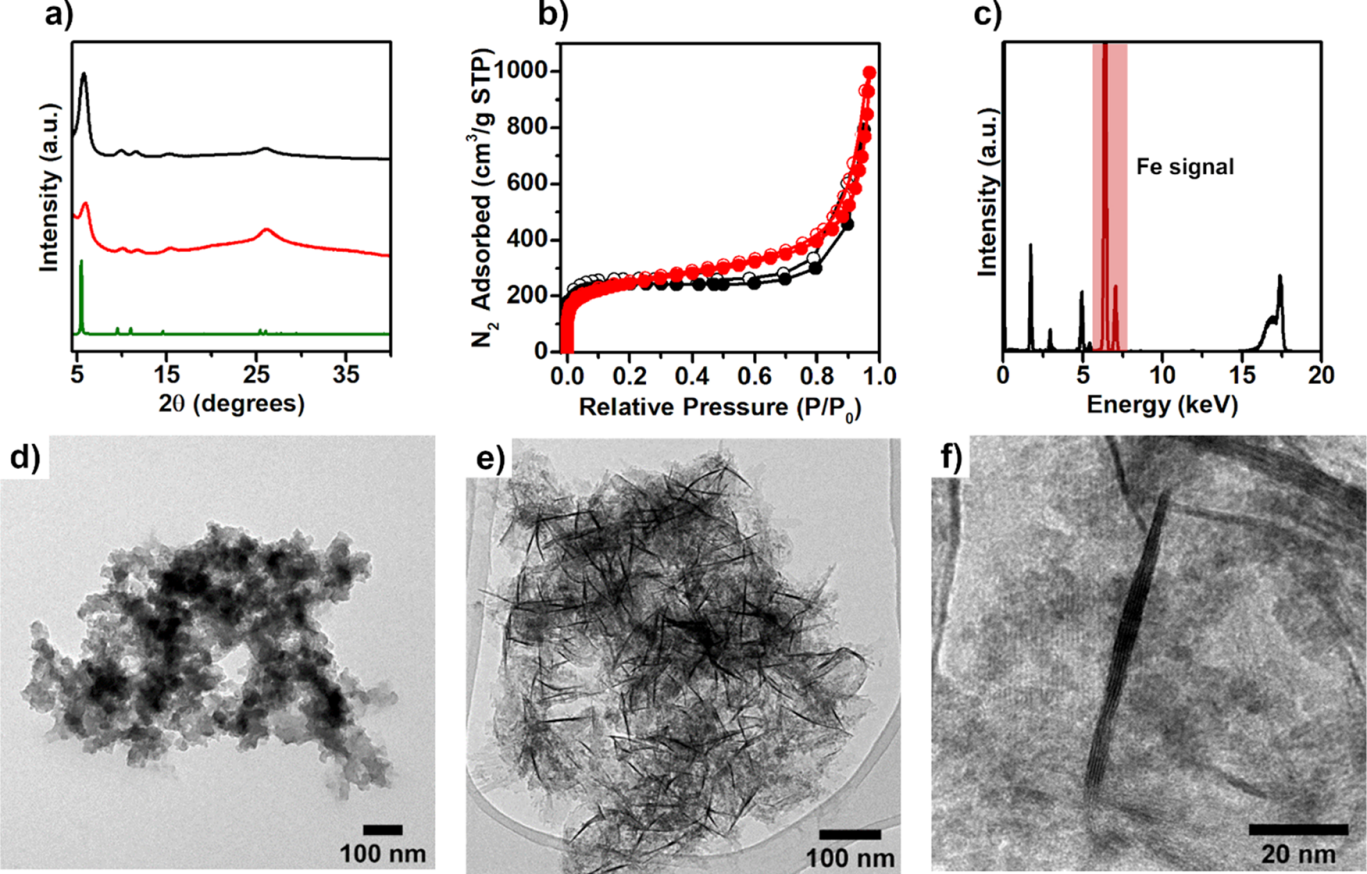
Nanocomposite characterization. (a) PXRD pattern of Tz-COF(s) (black), FeOOH@Tz-COF (red), and the theoretical pattern simulated for Tz-COF (green). (b) Nitrogen adsorption isotherm comparison between Tz-COF(s) (black) and FeOOH@Tz-COF (red). Filled dots: adsorption; empty dots: desorption. (c) Total reflection X-ray fluorescence (TXRF) spectrum of FeOOH@Tz-COF with the iron signal highlighted in red. (d) TEM image of Tz-COF(s). (e and f) TEM images of FeOOH@Tz-COF showing the distribution and size of the FeOOH nanorods.

The crystalline structure of Tz-COF(s) and FeOOH@Tz-COF was confirmed by powder X-ray diffraction (PXRD). Both materials have crystalline patterns matching the theoretical structure reported for this COF with an eclipsed AA stacking (Figure 2a).^26^ The PXRD pattern of Tz-COF(s) showed high crystallinity, which is especially remarkable as broad peaks are usually expected for nanoparticles with small crystalline domains.^27^ The FeOOH@Tz-COF pattern showed good crystallinity, although the peaks are slightly broader and have a higher background because of the incorporation of the iron oxyhydroxide. The increase in the relative intensity of the peak at 26° corresponding to the (001) reflection plane and associated with the interlayer distance can also be attributed to this matter. The absence of additional peaks indicates that the iron oxyhydroxide present in the composite does not present a long-range order (Figure S8, SI).

N_2_ adsorption isotherm analysis at 77 K was carried out to determine the permanent porosity of Tz-COF(s) and FeOOH@Tz-COF. The isotherms were adjusted to the Brunauer–Emmett–Teller (BET) theory and showed a surface area of 958 and 892 m^2^ g^–1^ (Figures S9–S12, SI), respectively (Figure 2b). The pore width distribution calculated reveals an average pore width of 1.4 nm for both samples (Figure S13, SI), which suggests that the iron oxyhydroxide is not located inside the pores of the COF in the composite because it does not block them.

Different techniques were used to study the presence of iron oxyhydroxide in FeOOH@Tz-COF. First, the amount of iron was evaluated with TXRF. A significant value of 27.2 wt % of Fe was found in the composite samples (Figures 2c, S14 and Table S2, SI). Thus, around 83% of the Fe atoms from the iron(II) sulfate used in the synthesis were successfully incorporated into the COF structure. These results agree with the elemental analysis measurements that show the carbon, nitrogen, and hydrogen percentages decrease substantially from Tz-COF(s) to FeOOH@Tz-COF because of the iron oxyhydroxide incorporation (see the Experimental Section).

The morphology of FeOOH@Tz-COF was evaluated by performing transmission electron microscopy (TEM) and scanning electron microscopy (SEM). For Tz-COF(s), TEM and SEM images showed a homogeneous aggregate of 20 nm COF nanoparticles (Figures 2d, S15 and S16, SI), which agrees with the size obtained by DLS measurements. As for FeOOH@Tz-COF, TEM and SEM images showed iron oxyhydroxide nanorods embedded between the nanoparticles producing a homogeneous nanocomposite (Figures 2e, S17 and S18, SI). The obtained nanorods display lengths from 40 to 90 nm, with width values between 1 and 6 nm (Figure 2f). These dimensions are much smaller than those typically reported for iron oxyhydroxides^28,29^ and are likely achieved thanks to a templating effect of Tz-COF during the growth of the nanorods, because blank experiments performed without the building blocks of Tz-COF (using just empty CTAB/SDBS micelles, acetic acid, and ammonia, see Section S1 Methods in the SI) yielded a dark brown iron oxyhydroxide without a defined morphology, as shown by SEM (Figure S19, SI). Furthermore, the nanorods exhibit a very homogeneous size distribution, ca. 75% showed lengths ranging from 50 to 80 nm, and 86% showed widths from 2 to 5 nm (Figure S20a and b, SI). In addition, there is a clear tendency to increase the width as the length increases (Figure S20c, SI). Regarding the distribution of the nanorods, the microscopy images show that they are well dispersed in the Tz-COF matrix and confirm that they are not located inside the Tz-COF pores, but rather in between Tz-COF particles.

**Figure 3:**
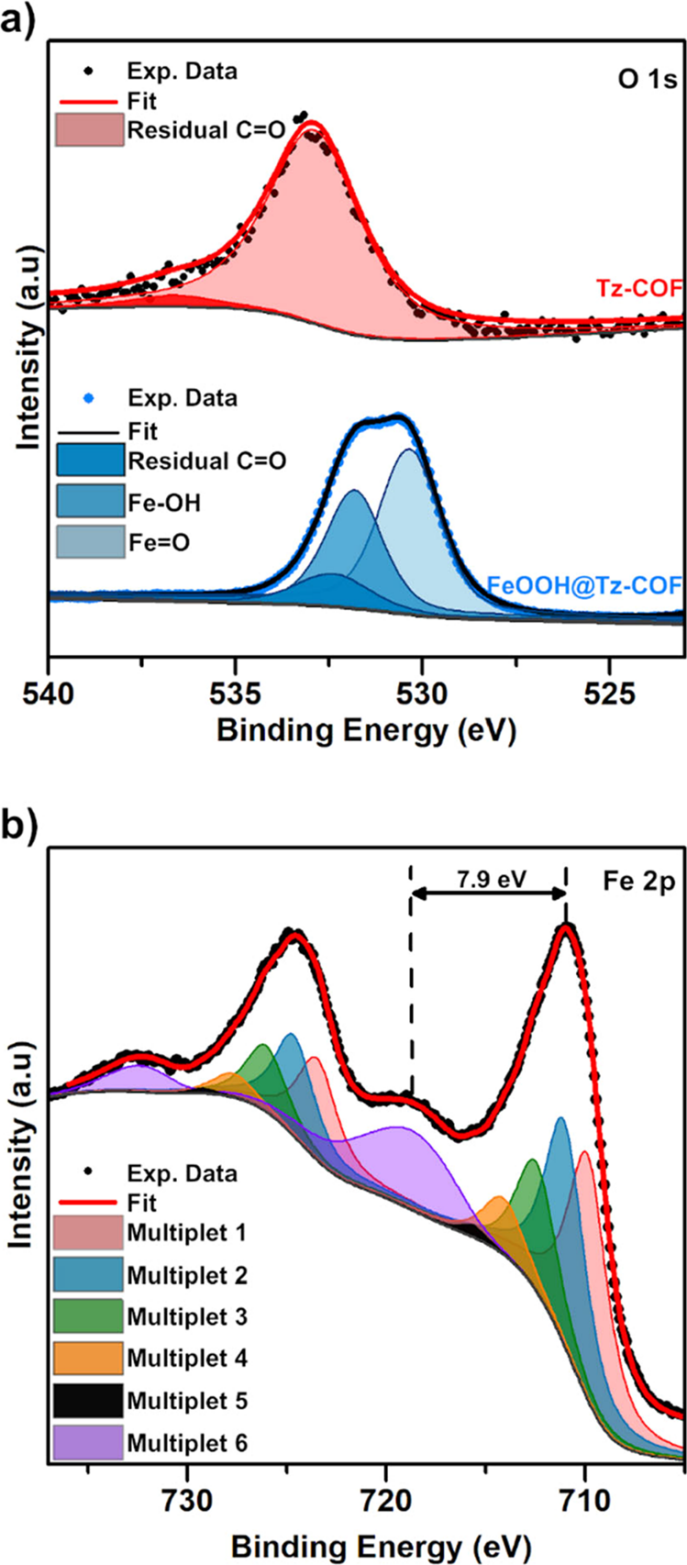
(a) XPS O 1s core-level spectra of Tz-COF(s) and FeOOH@Tz-COF, including a line shape analysis and deconvolution of the peaks. Note that the O 1s intensity of Tz-COF(s) is multiplied by 15, compared to FeOOH@Tz-COF. (b) XPS data for Fe 2p core level of FeOOH@Tz-COF. Deconvolution is based on ref (30)

X-ray photoemission spectroscopy (XPS) experiments have been performed on the Tz-COF(s) and FeOOH@Tz-COF samples to obtain further information on the nature of the iron oxyhydroxide nanorods formed. The O 1s core level analysis for Tz-COF(s) showed a low oxygen contribution and an almost single component attributed to the residual C=O groups from the aldehyde monomers. On the other hand, FeOOH@Tz-COF showed an intense O 1s core level with three components, a minor component attributed to the C=O group from Tz-COF(s), and two distinct main features that correspond to the ones expected for iron oxyhydroxides (Figures 3a and S22, SI). For the Fe 2p core level of FeOOH@Tz-COF, a line shape analysis was made according to the reported multiple peak parameters for lepidocrocite.^30^ In addition, the distance from the main peak to the satellite-2p^3/2^ was measured. The value of 7.9 eV found is associated with lepidocrocite, and the good fit of its components (Figures 3b and S23, SI) confirmed the nature of the nanorods as being the Fe(III) oxyhydroxide lepidocrocite (γ-FeOOH).

The capability of FeOOH@Tz-COF to capture As(III) ions from aqueous solutions was evaluated using inductively coupled plasma-mass spectrometry (ICP-MS) to measure the amount of As remaining in solution after incubation with FeOOH@Tz-COF. In a typical experiment, a solid sample of FeOOH@Tz-COF was placed in an aqueous solution (pH = 7) of As_2_O_3_. Then, the suspension was stirred for 3 h under ambient conditions and centrifuged at 6000 rpm to separate the supernatant from the material. As a first assessment of the As(III) removal capacity of FeOOH@Tz-COF, 600 mg L^–1^ of FeOOH@Tz-COF were used, leading to a 98.4% uptake of the 1.75 mg L^–1^ of As(III) in just 3 h. The affinity of the material for As(III) was estimated by the distribution coefficient (*K*_d_), and the value of 5.1 × 10^4^ mL g^–1^ obtained indicated the remarkable adsorption capacity of the material. The composite is a significantly better adsorbent than Tz-COF(s), which showed an As(III) uptake of just 19.1% and a value of *K*_d_ of 3.9 × 10^2^ mL g^–1^; this suggests that arsenic adsorption is mainly due to the iron oxyhydroxide nanorods. Tz-COF(s), on the other hand, seems to act as a porous matrix that prevents their aggregation and deactivation while allowing the arsenic solution to have access to the active sites in the surface of the nanorods.

To model the As(III) uptake capacity of FeOOH@Tz-COF, different experiments were performed for 3 h using aqueous solutions with initial arsenic concentrations varying from 0.5 to 120 mg L^–1^. The data were well-fitted with the Freundlich model (Figure S24, Table S3, SI), yielding a high correlation coefficient (*R*^2^ = 0.998) that suggested multilayer sorption and a heterogeneous surface in the material.^31^ The Freundlich isotherm can be expressed as eq 1:
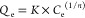
1where *Q*_e_ is the amount of arsenic adsorbed per unit weight of adsorbent (mg g^–1^), *C*_e_ is the equilibrium concentration of arsenic (mg L^–1^), *K* is the partition coefficient (L g^–1^), and 1/*n* determines the shape of the isotherm. One of the major disadvantages of the Freundlich equation is that it does not predict the maximum adsorption capacity of the material.^32^ Nevertheless, the obtained experimental value of *Q*_e_ of 272 mg g^–1^ is much higher than that of most of the materials reported so far at pH = 7. On the other hand, the value of 1/*n* obtained from the equation is found to be lower than 1 (1/*n* = 0.762), which is usually related to favorable adsorption at low As(III) concentrations.^31,33^ To test this limit, we used a very low concentration of 10 μg L^–1^ of As(III), the WHO limit for drinking water. FeOOH@Tz-COF showed an impressive capability even with a concentration of the composite of just 200 mg L^–1^, as it was capable of quantitatively removing 99.4% of the As(III), leading to a final concentration of 0.06 μg L^–1^. The great uptake capacity of the material is due to the efficient dispersion and high accessibility of the surface of the iron oxyhydroxide nanorods in the porous Tz-COF matrix, as high-surface area materials excel in adsorption–desorption processes.^34^

**Figure 4:**
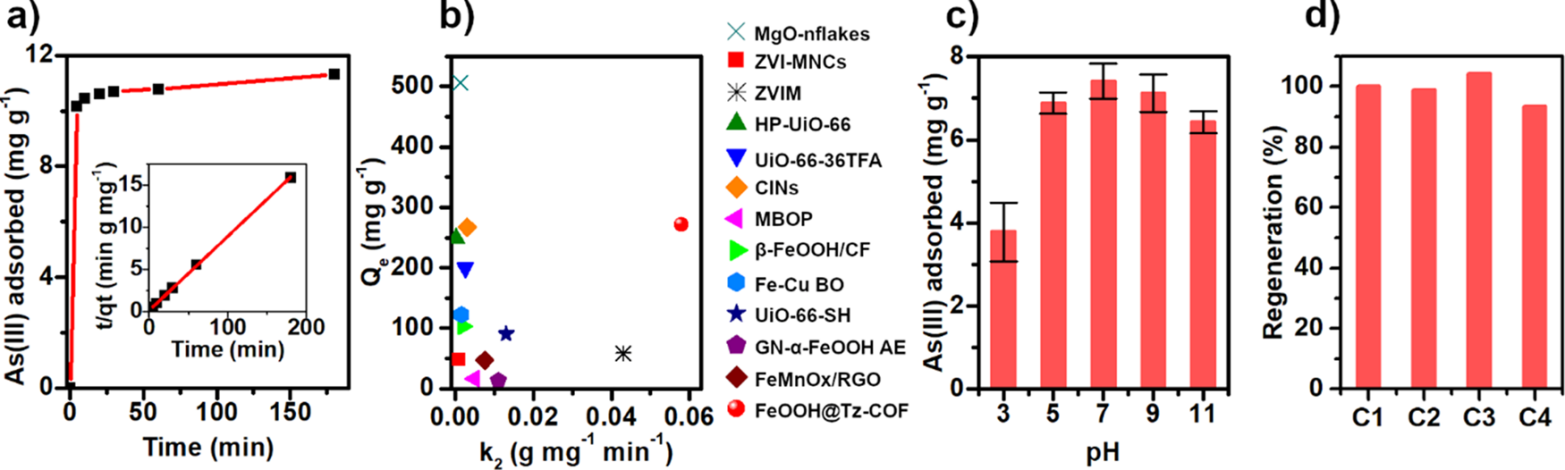
(a) Kinetic investigation of FeOOH@Tz-COF. The inset shows the pseudo-second-order kinetic plot for the adsorption. (b) Comparison scheme of the maximum retention capacity of As(III) and *k*_2_ of reported arsenic adsorbents: MgO-nflakes,^14^ ZVI-MNCs,^35^ ZVIM,^36^ HP-UiO-66,^10^ UiO-66-36TFA,^11^ CINs,^37^ MBOP,^38^ β-FeOOH/CF,^39^ Fe-Cu BO,^40^ UiO-66-SH,^41^ GN-α-FeOOH AE,^42^ FeMnOx/RGO,^43^ and FeOOH@Tz-COF (this work). (c) As(III) adsorbed by FeOOH@Tz-COF at different pH values. (d) Recyclability of FeOOH@Tz-COF for As(III) remediation in water.

Additionally, several experiments were carried out with an aqueous As(III) solution of 1.75 mg L^–1^ at different intervals to study the kinetics of the removal of arsenic ions by FeOOH@Tz-COF. The kinetic data can be fitted to a pseudo-second-order model typically used to describe the kinetics of heavy metal removal and expressed as eq 2:
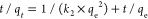
2where *q_t_* (g mg^–1^) is the amount of As(III) adsorbed at a time *t*, *k*_2_ is the rate constant of pseudo-second order (g mg^–1^ min^–1^), and *q*_e_ is the amount of arsenic adsorbed at equilibrium (g mg^–1^). An excellent value of *k*_2_ (0.058 g mg^–1^ min^–1^) and an extremely high correlation coefficient (*R*^2^ = 0.999) were obtained (Figure 4a). These results confirm the efficiency of FeOOH@Tz-COF in the elimination of arsenic in water and show that it is faster than many other arsenic adsorbents reported to this day. Figure 4b displays the maximum uptake and rate constant of the most remarkable materials for As(III) adsorption. It can be seen that even though magnesium oxide nanoflakes show higher values of maximum uptake,^13,14^ their adsorption kinetics are extremely slow; on the other hand, the FeOOH@Tz-COF nanocomposite displays an outstanding combination of fast kinetics and uptake capacity.

The effect of pH on arsenic uptake was studied to assess the chemical stability of the adsorbent. The adsorption capacity of FeOOH@Tz-COF was tested with 3 h experiments using 1.75 mg L^–1^ As(III) aqueous solutions with pH values from 3 to 11 (Figure 4c). The high removal observed in the range of pH 5–11 confirmed the efficiency under different conditions and its potential in water treatment in a wide range of conditions. An experiment assessing the interfering effect of other highly toxic metal ions was performed to study the influence of other ions on the As(III) adsorption capacity of the material. A 25 mL solution containing a mixture of As(III), Cd(II), Pb(II), and Hg(II) was treated for 3 h with FeOOH@Tz-COF and then centrifuged, and the extract was analyzed. The As(III) uptake in the presence of these ions was 81.2%, a result only slightly lower than the one obtained for the experiment performed under the same conditions with only an As(III) solution (85.1%). FeOOH@Tz-COF was also capable of adsorbing significant amounts of Pb(II) and Hg(II) (Table S7, SI), but the saturation experiments with these cations showed a poor fit to Langmuir and Freundlich isotherms and a lower uptake capacity than other materials already reported.^21,44^ Therefore, FeOOH@Tz-COF can effectively eliminate As(III) of polluted water in the presence of other heavy metals, even performing a noncompetitive uptake of Pb(II) and Hg(II). On the other hand, the interference of phosphates with As(III) adsorbents is well known.^45^ To investigate this matter, another experiment was proposed using anions like PO_4_^3–^, Cl^–^, SiO_3_^2–^, and SO_4_^2–^. Indeed, only PO_4_^3–^ affected the elimination of As(III) in water by reducing the uptake to 66.7% (Table S7, SI).

Finally, the regeneration of the FeOOH@Tz-COF adsorbent by releasing the captured As(III) was one of the challenges needed to be solved to use the composite for water remediation. For this purpose, used FeOOH@Tz-COF was treated with an alkaline solution following a general method for arsenic desorption.^46,47^ After stirring the nanocomposite in a 0.1 mol L^–1^ NaOH solution overnight, it was washed with water and ethanol and activated by supercritical drying. The material maintained its ability to capture As(III) (Figure 4d) and its crystallinity (Figure S26, SI) even after four cycles. Additionally, TEM images of the material after the regeneration cycles showed that the lepidocrocite nanorods were not affected by the adsorption–desorption processes and maintained their distribution and morphology (Figure S27, SI). These results indicate the structural robustness of the material even under harsh conditions and repeated use.

## Conclusions

The use of Tz-COF nanoparticles has successfully enabled preparation of a novel iron oxyhydroxide@COF nanocomposite by templating the growth of lepidocrocite nanorods. Thanks to the synergistic behavior of the COF structure acting as a porous matrix that maintains a homogeneous dispersion of the FeOOH nanorods, which are known to perform extremely well in As(III) capture, the FeOOH@Tz-COF nanocomposite excels at As(III) removal. It combines an extremely high As(III) adsorption capacity with one of the fastest removal rates in a wide pH range, making it a very attractive material for use in water remediation. This work shows the potential of COF nanocomposites and the control of morphology, particle size, and distribution that can be achieved by the use of COF nanoparticles, which will prove central in the preparation of COFs for multiple applications.

## Experimental Section

### Materials

1,3,5-Benzenetricarboxaldehyde and 2,4,6-tris(4-aminophenyl)-1,3,5-triazine were purchased from Sigma-Aldrich and Fluorochem, respectively. Other chemicals and solvents were purchased from Sigma-Aldrich and used without further purification unless specified.

### Synthesis of Tz-COF Colloidal Nanoparticle Solution and Flocculation

21.6 mg of 2,4,6-tris(4-aminophenyl)-1,3,5-triazine (Tz) (0.0615 mmol) are dissolved in 0.125 mL of DMSO. The solution is added dropwise to 29 mL of a 0.1 mol L^–1^ aqueous solution of CTAB under sonication. Then, 0.9 mL of a 0.1 mol L^–1^ aqueous solution of SDBS are added dropwise and under sonication. Subsequently, 10 mg of 1,3,5-benzenetricarbaldehyde (0.0615 mmol of BTCA) are dissolved in 0.125 mL of DMSO. This solution is added dropwise to 29 mL of a 0.1 mol L^–1^ aqueous solution of CTAB under sonication. The suspension that appears quickly disappears. Then, 0.9 mL of a 0.1 mol L^–1^ aqueous solution of SDBS are also added dropwise and under sonication. The two resulting aqueous solutions are mixed, and 2.9 mL of acetic acid are added. A completely yellow solution is formed. The mixture is then deoxygenated performing four vacuum-argon cycles and allowed to react at 30 °C for 72 h. After the reaction, a completely transparent yellow colloidal solution is formed. The flocculation of the nanoparticles is carried out by adding 30% ammonia solution slowly up to pH 7 and then 100 mL of ethanol. The resulting suspension is centrifuged for 3 min at 1500 rcf, and the supernatant was removed. This washing procedure is repeated eight times. Finally, the sample was activated by critical point drying, and 26.4 mg of yellow solid were obtained (86.5%). Elemental analysis of Tz-COF: calculated for C_30_H_18_N_6_(H_2_O)_1.9_: C: 72.54%; H: 4.42%; N: 16.92%. Experimental: C: 73.7%; H: 4.55%; N: 15.66%.

### Synthesis of the FeOOH@Tz-COF Nanocomposite

The colloidal solution of the Tz-COF prepared following the procedure above was treated with 2.5 mL of an aqueous solution of FeSO_4_·7H_2_O (0.05 g, 0.18 mmol). The reaction was kept at 30 °C for another 24 h. To achieve the flocculation of the nanoparticles, a 30% ammonia solution was added slowly until pH = 7 to the suspension and then 100 mL of ethanol. With the neutralization, a change in the color is observed, and the solution turns from yellow to brown. The suspension was centrifuged for 3 min at 1500 rcf, and the supernatant was removed. This washing procedure is repeated eight times. Finally, the sample was activated by critical point drying, and 35.9 mg of a brown solid were obtained. Experimental elemental analysis: C: 43.08%; H: 3.94%; N: 9.42%.

### As(III) Uptake Experiments

The As(III) uptake was performed by mixing the solid sorbent with arsenic solutions of concentrations from 0.5 to 120 mg L^–1^ with agitation at 300 rpm for 3 h. Centrifugation (four cycles of 5 min at 6000 rpm) was then performed to guarantee the total isolation of the adsorbent from the solution after treatment. Then the arsenic concentration solutions were determined using ICP-MS. The adsorptive capacity was calculated by comparing the difference in As(III) concentration between the original solutions and the samples after treatment.

### Kinetic Study of As(III) Uptake

The arsenic removal rate study was performed by mixing the nanocomposite with different arsenic solutions of 1.75 mg L^–1^ with agitation at 300 rpm. Each experiment was performed for different periods, from 5 min to 3 h. Centrifugation was then performed following the same procedure as above, and the arsenic concentration of the supernatant was determined using ICP-MS. The adsorptive capacity was calculated by comparing the difference in As(III) concentration between the original solutions and the samples after treatment.

### Effect of the pH Studies

The effect of pH on the arsenic removal was assessed by mixing the nanocomposite with different arsenic solutions of 1.75 mg L^–1^ with pH values from 3 to 11 with agitation at 300 rpm for 3 h. Centrifugation was then performed following the same procedure as above, and the arsenic concentration of the supernatant was determined using ICP-MS. The adsorptive capacity was calculated by comparing the difference in As(III) concentration between the original solutions and the samples after treatment.

### Interference Experiments

The ion uptakes from aqueous solutions were studied using the batch method. The metal ions involved are used as their nitrate salts and the anions as their sodium salts. After mixing the solid sorbents with the solutions for 3 h, centrifugation was performed under the same conditions as the arsenic uptake experiments. The ion concentrations in samples after treatment were determined using ICP-MS, and the adsorptive capacity was evaluated from the difference in the concentration of the ions between the original and the treated solutions.

### Regeneration Studies

A sample of 15 mg of FeOOH@Tz-COF was treated with a solution of 1.75 mg L^–1^ of As(III) (25 mL) at room temperature. After stirring for 3 h, the solid was filtered, and the supernatant was analyzed with ICP-MS. The material was treated with 30 mL of a NaOH (0.1 M) solution and stirred overnight at 300 rpm. Then the material was washed several times, first with deionized water until pH = 7 and then with ethanol. Finally, the sample was activated by critical point drying. The same procedure was repeated for four cycles.

## Supporting Information Available

Methods, synthesis of iron oxyhydroxide in a micellar system without COF building blocks; DLS of the nanoparticle suspension; FT-IR and ^13^C CP-MAS NMR spectra; TGA profiles; PXRD patterns; BET plot for N_2_ sorption and pore size distribution; TXRF spectrum; TEM and SEM images and EDX spectra; statistical study of lepidocrocite nanorods; complete characterization by XPS; and tables for the values of concentration by ICP-MS obtained in the experiments for the elimination of As(III) in water (isotherm, kinetic, pH selectivity, regeneration, and comparison with other materials) (PDF)
