# Supplementary material for: Iron Oxyhydroxide-Covalent Organic Framework Nanocomposite for Efficient As(III) Removal in Water
Source: ACS Appl Mater Interfaces. 2022 Oct 25;14(44):50163–70. doi: 10.1021/acsami.2c14744 (PMC9827450; doi:10.1021/acsami.2c14744)
Supplement: Supplementary file 1 — am2c14744_si_001.pdf [file am2c14744_si_001.pdf]

Supporting Information

Iron oxyhydroxide-covalent organic framework nanocomposite for efficient As(III) removal in water

Ana Guillem-Navajas,[1] Jesús Á. Martín-Illán,[1] Elena Salagre,[2] Enrique G. Michel,[2,3] David Rodriguez-San-Miguel[1] and Félix Zamora*,[1,3]*

[1] Departamento de Química Inorgánica, Facultad de Ciencias, and Institute for Advanced Research in Chemical Sciences (IAdChem) and Condensed Matter Physics Institute (IFIMAC). Universidad Autónoma de Madrid, 28049 Madrid, Spain.
[2] Departamento de Física de la Materia Condensada. Facultad de Ciencias. Universidad Autónoma de Madrid, Madrid 28048, Spain.
[3] Condensed Matter Physics Center (IFIMAC), Facultad de Ciencias, Universidad Autónoma de Madrid, Madrid 28048, Spain.

Corresponding Author

E-mail: david.rodriguezs@uam.es, felix.zamora@uam.es

Index

Section S1. Methods..3
Section S2. Synthesis and characterization...6
Section S3. Elimination of As(III) in water with FeOOH@Tz-COF nanocomposite..21
References..26

Section S1. Methods

Powder X-ray Diffraction (PXRD)

Standard PXRD patterns were collected with a Bruker D8 Advance A25 X-ray powder diffractometer (CuKα1 radiation; λ = 1.5406 Å) equipped with a PSD-XE detector energy-discriminator (DAVINCI). Samples were mounted on a flat glass sample plate. Patterns were collected in the 3.5< 2θ<40º range with a step size of 0.03º and an exposure time of 1.3 s/step.

Fourier Transform Infrared Spectroscopy (FT-IR)

FT-IR spectroscopy was performed using a Perkin Elmer Spectrum 100 with a PIKE Technologies MIRacle Single Reflection Horizontal ATR accessory having a spectral range of 4000-650 cm^{-1}.

Elemental Analysis (EA)

Elemental analyses were obtained using LECO CHNS-932 elemental analyzer.

Solid-State ^{13}C CP-MAS Nuclear Magnetic Resonance Spectroscopy

Solid-State NMR spectra were recorded at room temperature on a Bruker AV 400 WB spectrometer using a triple channel, 4 mm probe with zirconia rotors, and a Kel-F cap. Cross-polarization with Magic Angle Spinning (CP-MAS) was used to acquire ^{13}C data at 100.61 MHz. The spectral width of the pulse sequence was 35 kHz, and the ^{1}H excitation pulse was 3 μs. The CP time was 3.5 ms. High power two-pulse phase modulation (TPPM) 1H decoupling was applied during data acquisition using a decoupling frequency of 80 kHz. Recycle delays were 4 s, and the sample spinning rate was 10 kHz.

Dynamic Light Scattering (DLS)

DLS measurements were carried out using a Vasco 1 particle size analyzer from Cordouan Technologies. Measurements were carried out at 90º scattering angle, 35 ºC, and the correlation function was collected for 20 s. A statistical analysis was performed in which 100 measurements were taken for each sample. The software NanoQ was used to process the data with the Padé-Laplace algorithm to obtain the hydrodynamic diameter.

Gas Adsorption

N_2 adsorption and desorption isotherms were performed at 77 K using a Micrometrics ASAP 2020 Plus analyzer. Before the measurement, samples were heated at 323 K overnight and outgassed at 10^{-6} Torr.

Critical point drying

The solid, immersed in ethanol, was transferred into a piece of dialysis tubing (Spectra/Por 1, MWCO: 6-8 kD) and then sealed. The membrane was then introduced into an SPI-DRY Critical Point Dryer – Jumbo and the chamber containing the sample was filled with liquid CO_2 at 10 ºC. After allowing it to exchange for 1 h the chamber was flushed with fresh liquid CO_2. This solvent exchange procedure was performed a total of 5 times. After the final exchange, the temperature was raised to 40 ºC to exceed the critical point of CO_2. Once this temperature was reached and CO_2 was in a supercritical state, the chamber was slowly vented at a rate of 10 bar h^{-1} until atmospheric pressure was reached and the sample could be recovered.

Thermogravimetry (TGA)

Thermogravimetric analyses of samples were performed using a Thermobalance TGA Q-500 thermal gravimetric analyzer from TA Instruments, with samples held in an aluminum pan under a nitrogen atmosphere. The samples were heated at 10 K min^{-1} within a temperature range of 25-1000ºC.

Scanning Electron Microscopy (SEM)

Scanning electron microscopy images were collected on a JEOL JSM 6335F scanning electron microscope at an acceleration voltage of 15 kV. Samples were dispersed over a slice of conductive adhesive adhered to a flat copper platform sample holder and then coated with gold using a Quorum Q150T-S sputter coater.

Transmission Electron Microscopy (TEM)

Transmission electron microscopy images were obtained using a JEOL-JEM 1400 equipped with an SDD detector. The accelerating voltage was set to 40 kV to minimize sample damage. Images were acquired using a high-resolution CCD camera (2048 x 2048 pixels). The samples were prepared by casting a drop of the material dispersed in water on a lacey carbon TEM grid.

Total Reflection X-Ray Fluorescence (TXRF)

TXRF analysis was carried out to analyse the content of iron in the sample. The measurements were performed with a Bruker TXRF S2 PicoFox Spectrometer with Mo

K radiation at 50 kV and 600 mA, an acquisition time of 500 s, and 10 ppm of vanadium, the internal standard. Samples were digested by microwave-assisted acid digestion with 3 mL of HNO_3 and 0.50 ml of HCl. The reactors were introduced in an UltraWAVE digestion system from Millestone (Italy) and heated up to 240 °C for 20 minutes with a base working pressure of 40 bars. All the chemicals used were Suprapur® grade acids from Fisher Scientific™. Ultrapure water with minimum resistivity of 18.2 MΩ (Milli-Q Element) was used.

X-ray photoelectron spectroscopy (XPS)

XPS analyses of COFs were performed on ground powders using a VG Escalab 200 R equipped with a hemispherical detector with 5 channeltrons (Pass energy: 2-200 eV) and a monochromatic Al Kα X-ray source (1486.61 eV). An initial analysis of all the elements present was carried out (wide scan: step energy 1 eV, dwell time 0.1 s, pass energy 50 eV), then a detailed analysis was performed (detail scan: step energy 0.1 eV, dwell time 0.1 s, pass energy 20 eV) with an angle of 90º for the electrons exit (normal emission). The spectra were processed using Igor Pro 6.3.7.2-WaveMetrics. Inc. software. The line shape of core levels was fitted using a Shirley background and asymmetric singlet pseudo-Voigt functions. The fit was optimized using a Levenberg-Marquardt algorithm with a routine running in IGOR Pro (WaveMatrix Inc.).[1] The quality of the fit was judged from a reliability factor, the normalized χ^2. Before the XPS measurements, the crystalline powders were pressed on copper foil.

Inductively coupled plasma-mass-spectrometry (ICP-MS)

The ICP-MS system used in this study was a quadrupole spectrometer Perkin-Elmer NexION 300XX, equipped with a collision/reaction cell to eliminate interferences. Blank standards and samples were prepared using Milli Q water in HNO3 1% (v/v) medium. The samples were analyzed with a 1/10 dilution.

Iron oxyhydroxide synthesis in a micellar system without Tz-COF nanoparticles.

58 mL of a 0.1 M aqueous solution of CTAB is mixed with 1.8 mL of a 0.1 M aqueous solution of SDBS under sonication. Subsequently, 2.9 mL of acetic acid are added, and the mixture is deoxygenated by performing 4 vacuum-argon cycles. Then, 2.5 mL of an aqueous solution of $FeSO_4{\cdot}7H_2O$ (0.05g, 0.18 mmol) is added and allowed to react at 30 °C for 24 h. To achieve the flocculation of the nanoparticles, a 30 % ammonia solution is added slowly till pH=7 to the suspension and then 100 mL of ethanol absolute. With the neutralization a change in the colour is observed, and the solution turns brown. The solid is centrifuged for 3 minutes at 1500 rcf and the supernatant removed. This washing procedure is repeated 5 times, and the sample is filtered and dried.

Section S2. Synthesis and characterization

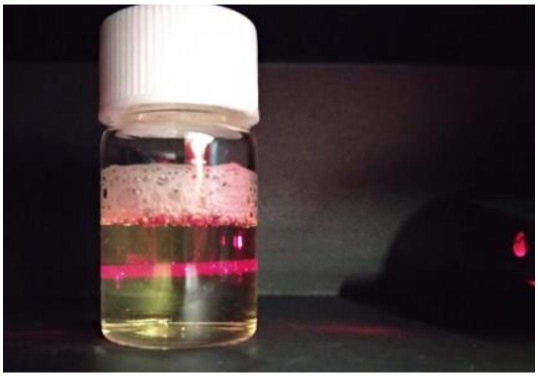

Figure S1. Tyndall effect in a transparent and homogeneous Tz-COF colloidal solution upon irradiation with laser light at 630 nm.

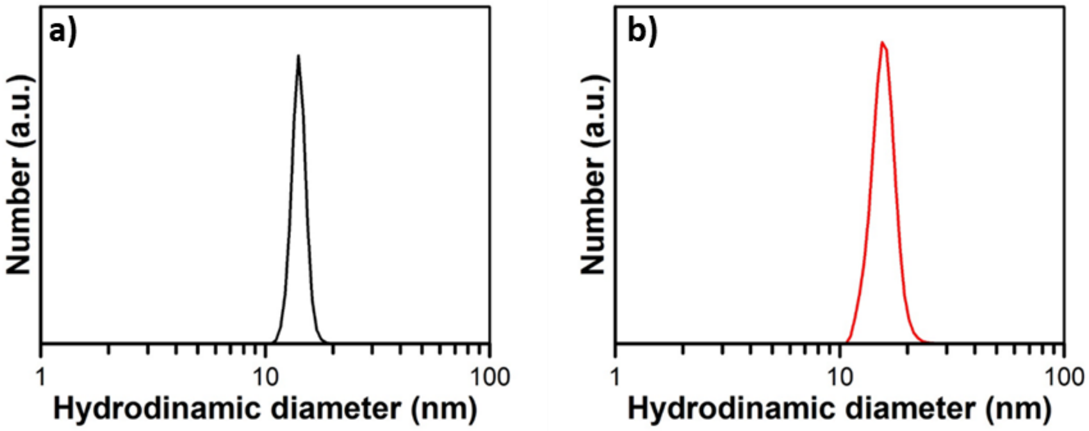

Figure S2. Size distributions obtained by DLS of the Tz-COF colloidal solution after 72 h (a) and the Tz-COF colloidal solution after the addition of $FeSO_4 \cdot 7H_2O$ (b).

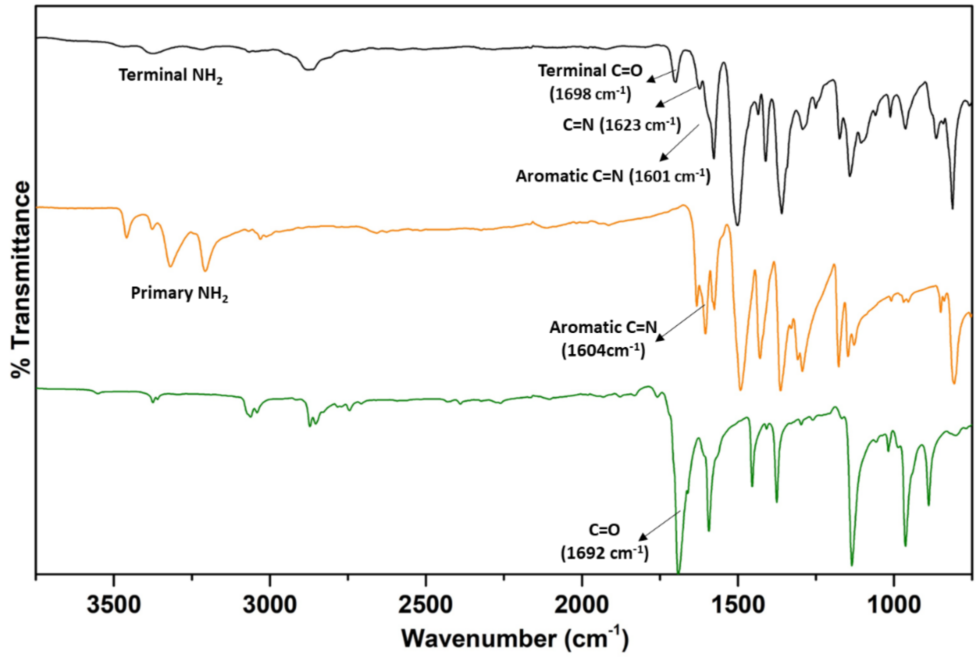

Figure S3. Comparative FTIR spectra of Tz-COF (black) and its monomers: Tz (yellow) and BTCA (green). The stretching bands at 1623 cm^{-1} and 1601 cm^{-1} correspond to C=N imine bond and aromatic triazine C=N bonds, respectively. The bands present at 1698 cm^{-1} (C=O str.) and 3400 cm^{-1} (N-H str.) indicate the presence of terminal aldehyde and amine groups.

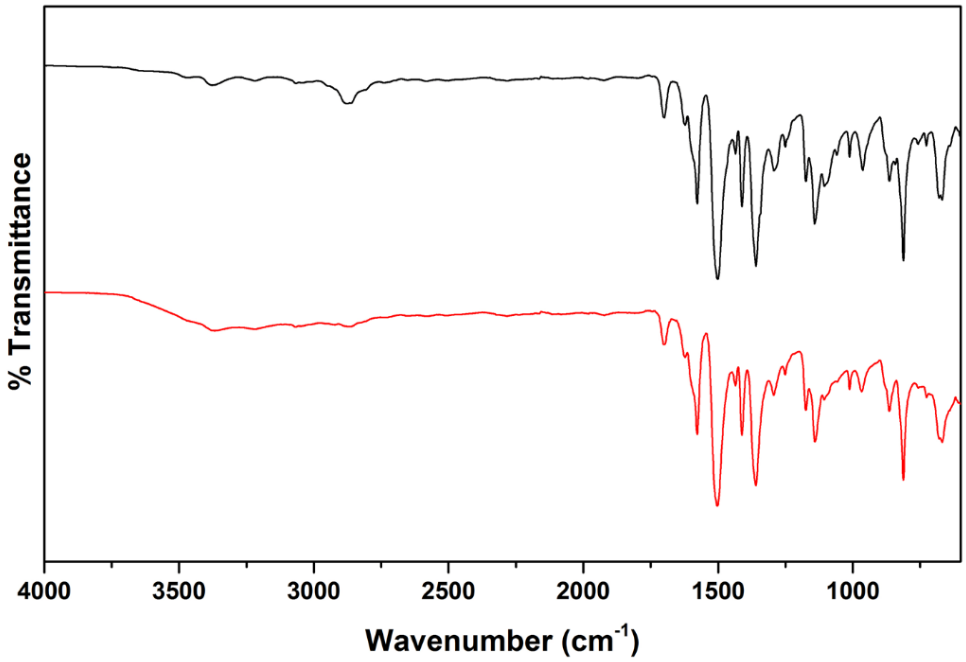

Figure S4. Comparative FTIR spectra of Tz-COF (black) and FeOOH@Tz-COF (red).

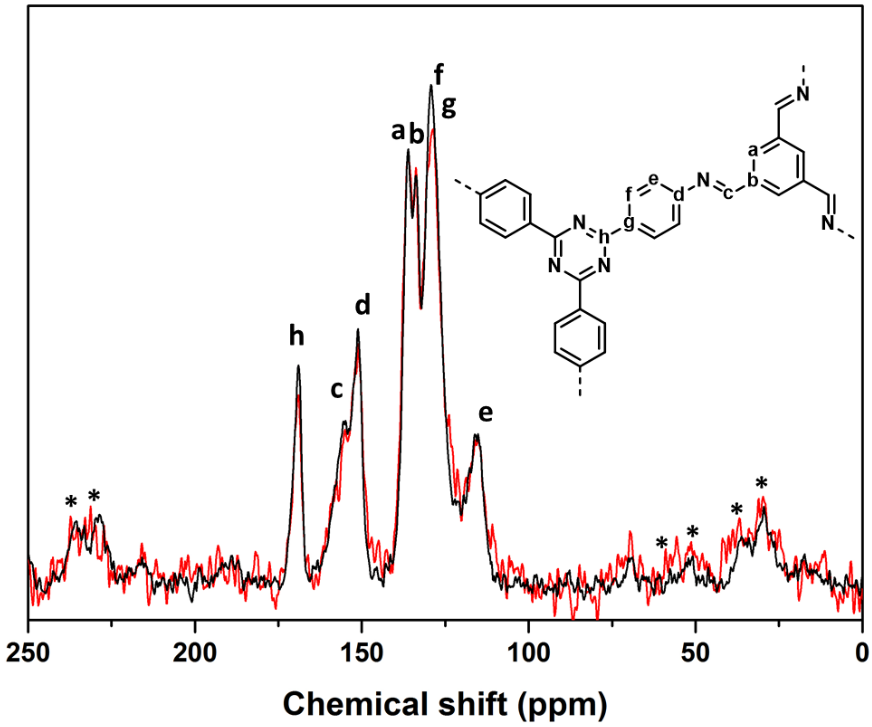

Figure S5.^{13}C CP-MAS NMR spectra of Tz-COF (black) and FeOOH@Tz-COF (red). The signal assignments are displayed on the fragment of the structure. Asterisks denote spinning sidebands.

Table S1. ^{13}C NMR spectra peak assignment.

Assignment	Signal (ppm)
a,b	136.10
c	154.60
d	151.19
e	116.10
f,g	129.22
h	168.99

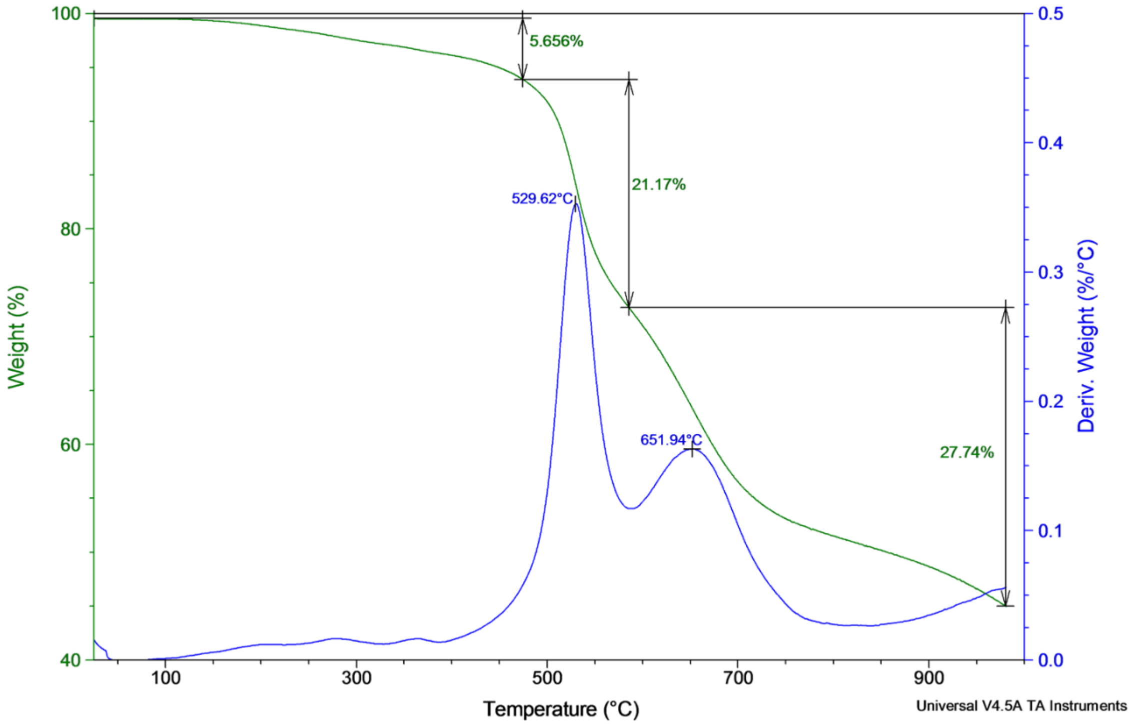

Figure S6. TGA profile of Tz-COF.

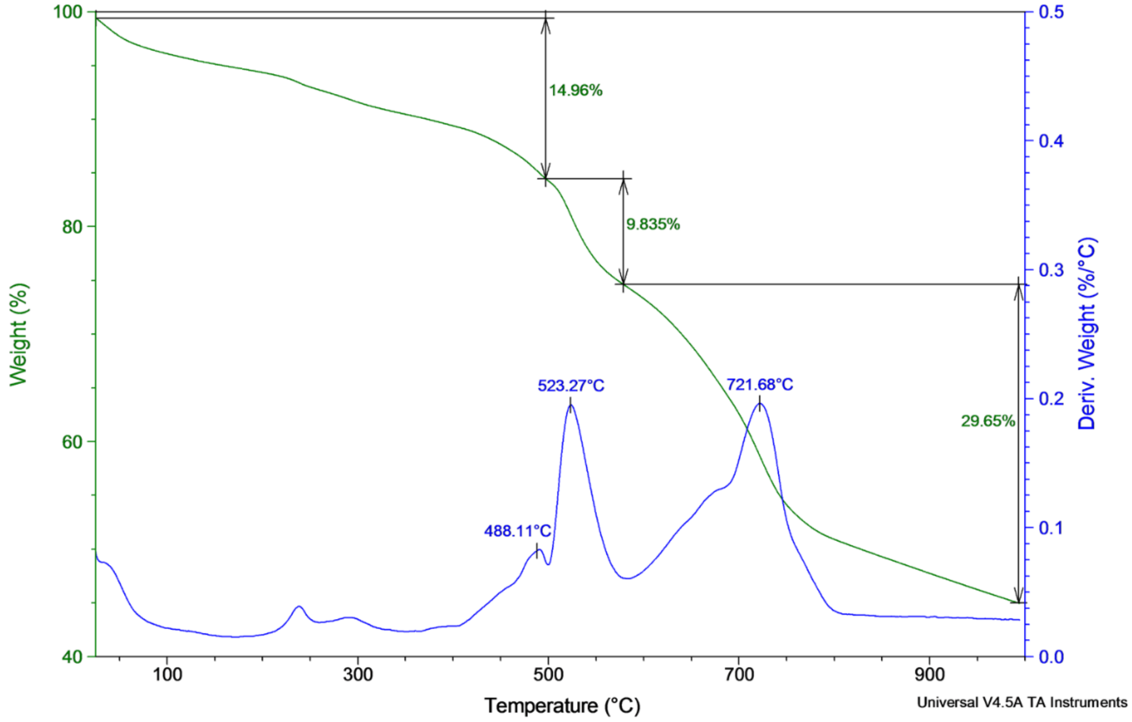

Figure S7. TGA profile of FeOOH@Tz-COF.

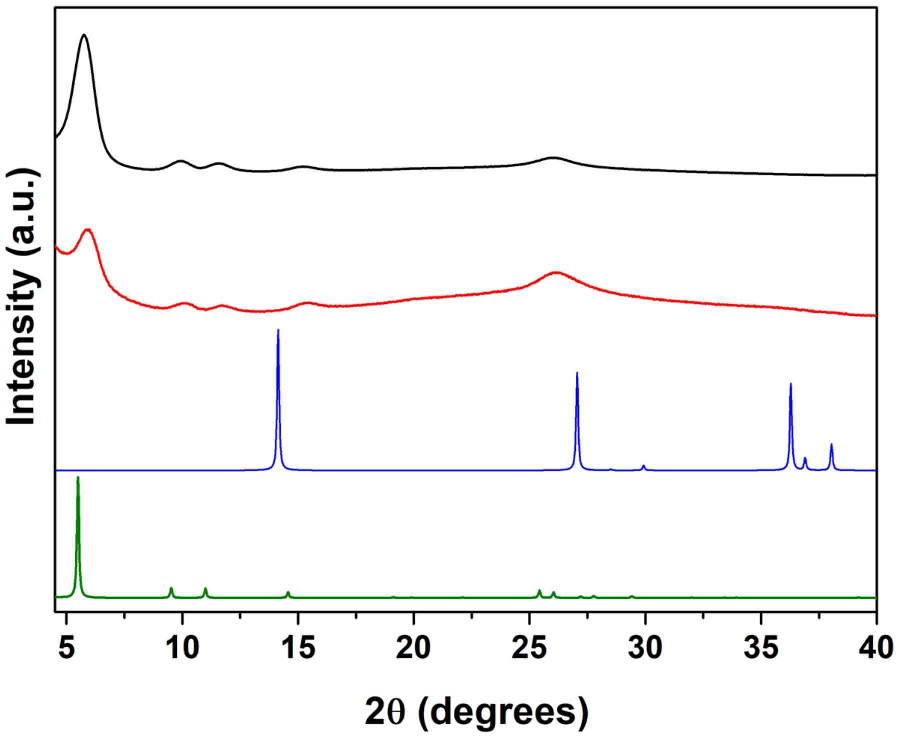

Figure S8. PXRD patterns of Tz-COF(s) (black), FeOOH@Tz-COF (red), simulated lepidocrocite PDF Card 00-044-1415 (blue), and simulated Tz-COF (green).

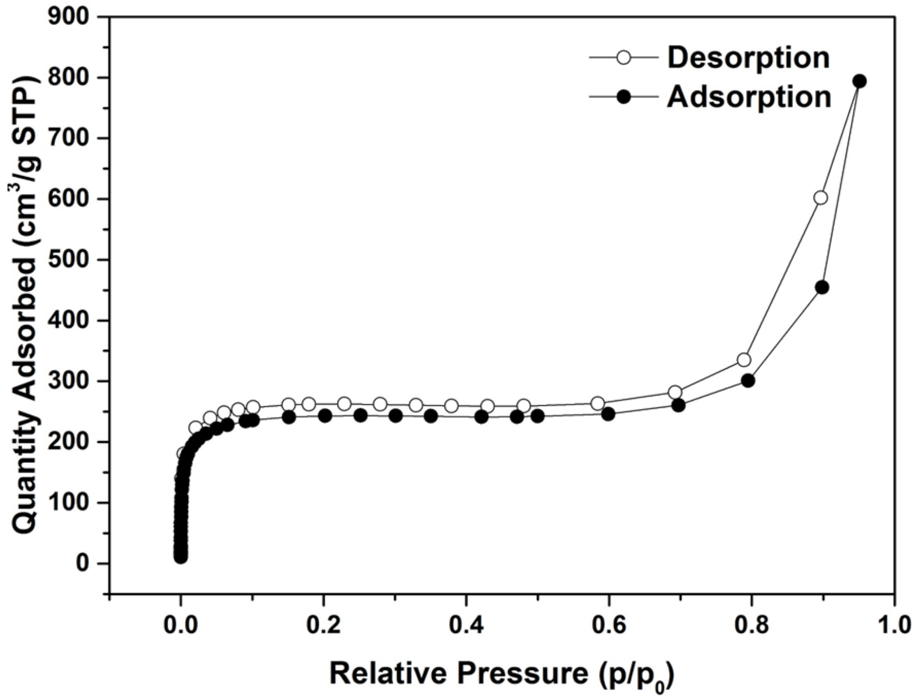

Figure S9. N_2 adsorption isotherm of Tz-COF(s).

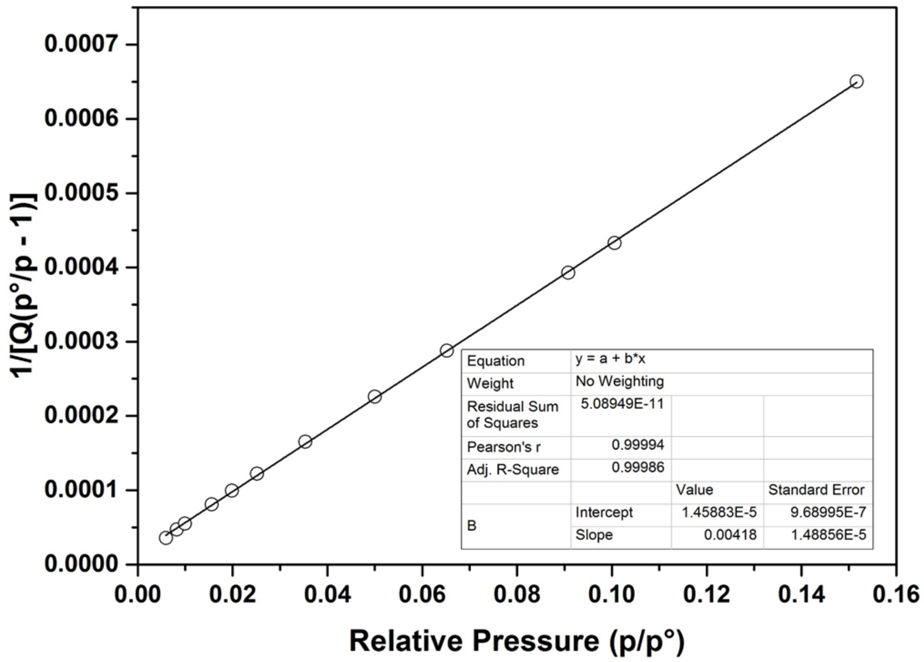

Figure S10. BET plot for N_2 sorption in Tz-COF(s).

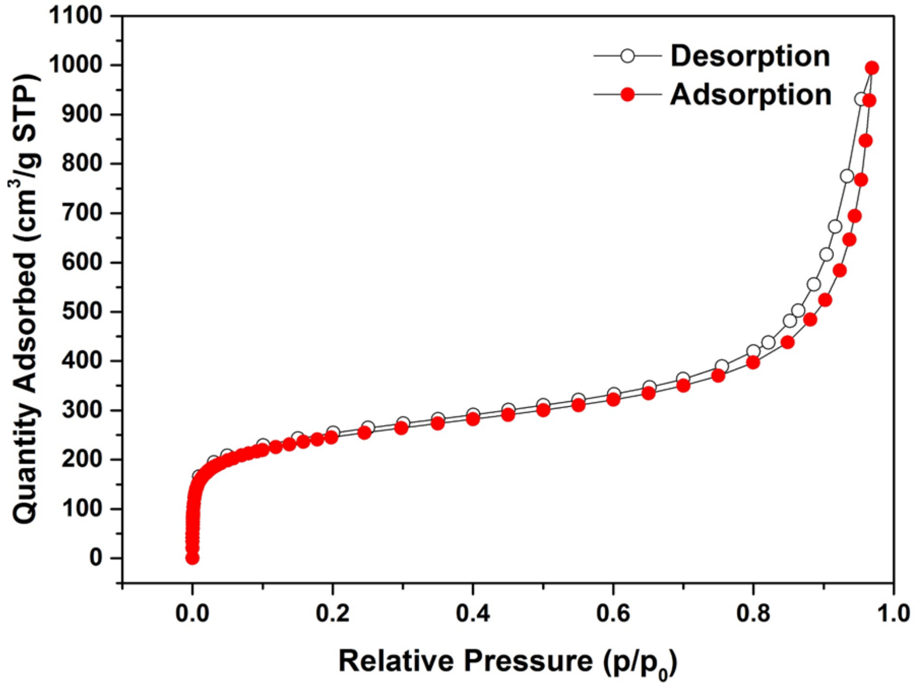

Figure S11. N_2 adsorption isotherm of FeOOH@Tz-COF.

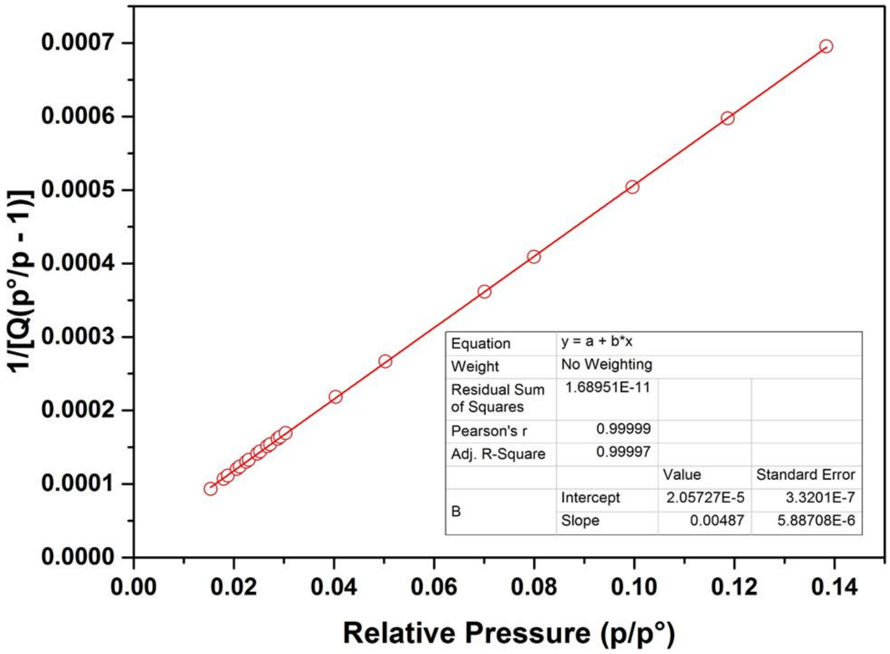

Figure S12. BET plot for N_2 sorption in FeOOH@Tz-COF.

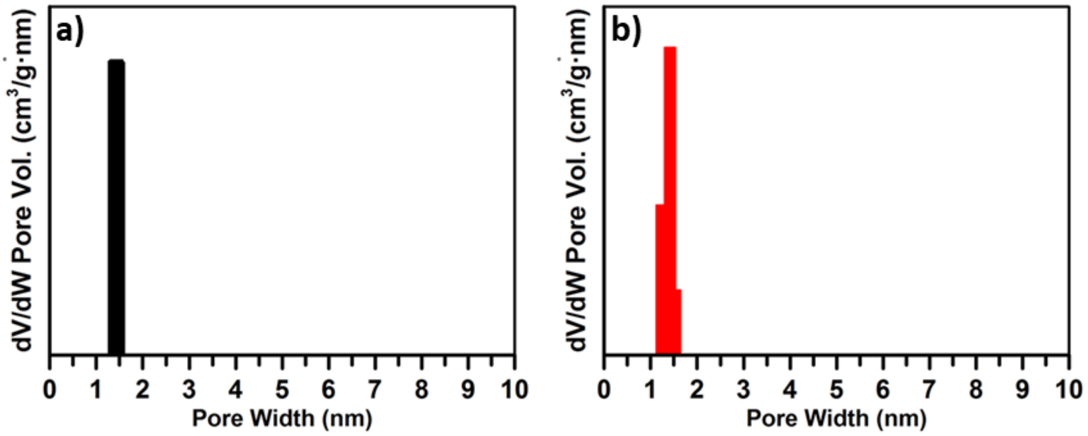

Figure S13. Pore size distribution of Tz-COF(s) (a) and FeOOH@Tz-COF (b).

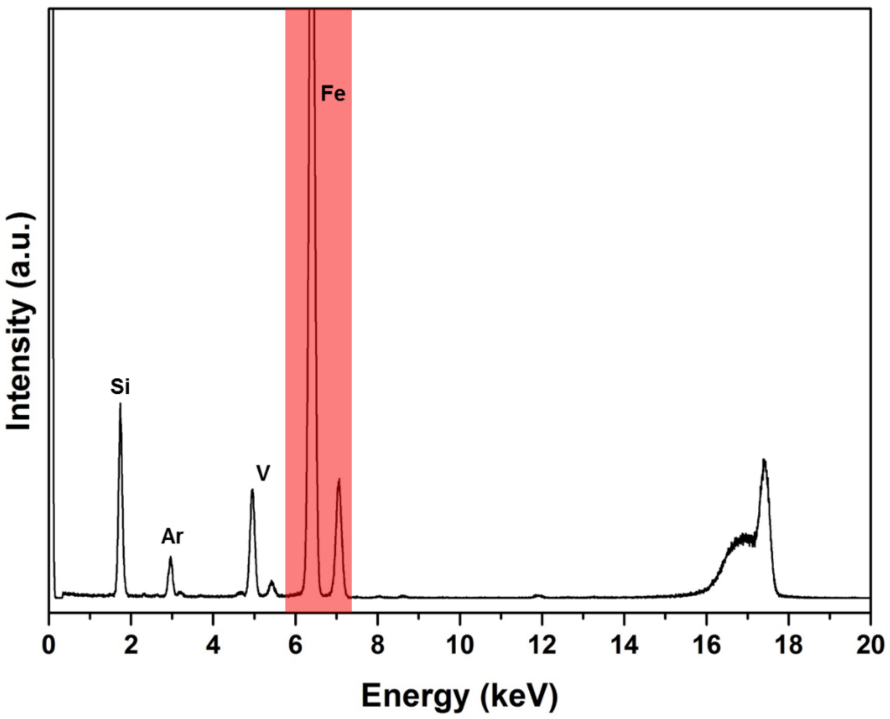

Figure S14. TXRF spectrum of FeOOH@Tz-COF. The Ar signal corresponds to the atmospheric Ar, the Si signal corresponds to the sample holder reflector, and V is the internal standard used in the measurement.

Table S2. TXRF data of FeOOH@Tz-COF. Si and V signals correspond to the sample holder and the internal standard, respectively.

Element	Line	Conc. (mg L^{-1})	Sigma (mg L^{-1})	RSD (%)	%wt
Si (SH)	K12	504.8	4.0	0.8	276.36
S	K12	0.86	0.19	21.7	0.471
Cl	K12	0.45	0.11	25.3	0.246
V (IS)	K12	10000	0.09	0.9	5474
**Fe**	**K12**	**49.74**	**0.20**	**0.4**	**27.23**
Cu	K12	0.048	0.004	7.8	0.026
Zn	K12	0.035	0.003	9.0	0.019
Br	K12	0.050	0.003	5.0	0.027

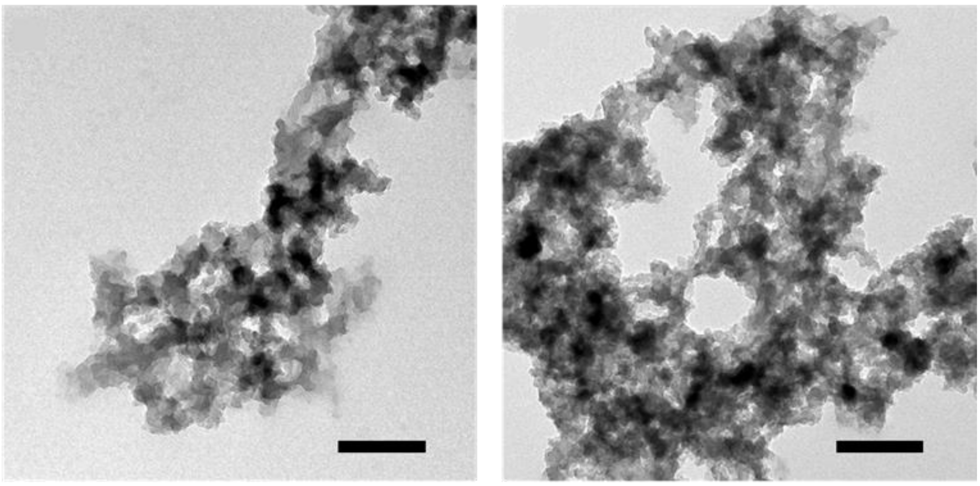

Figure S15. TEM images of Tz-COF(s). Scale bar equals 200 nm.

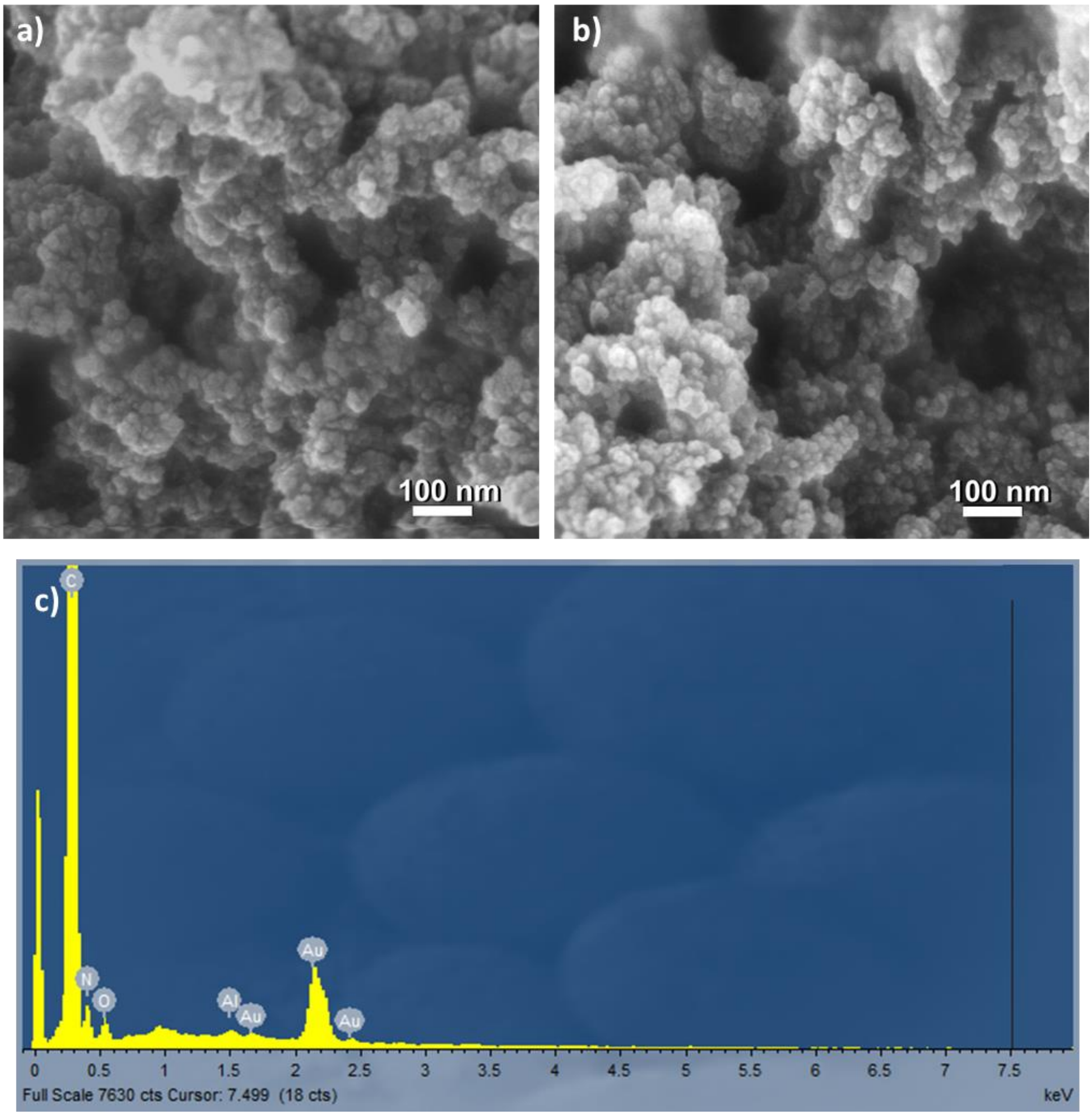

Figure S16. SEM images of Tz-COF(s) (a-b) and EDX spectrum of the material (c).

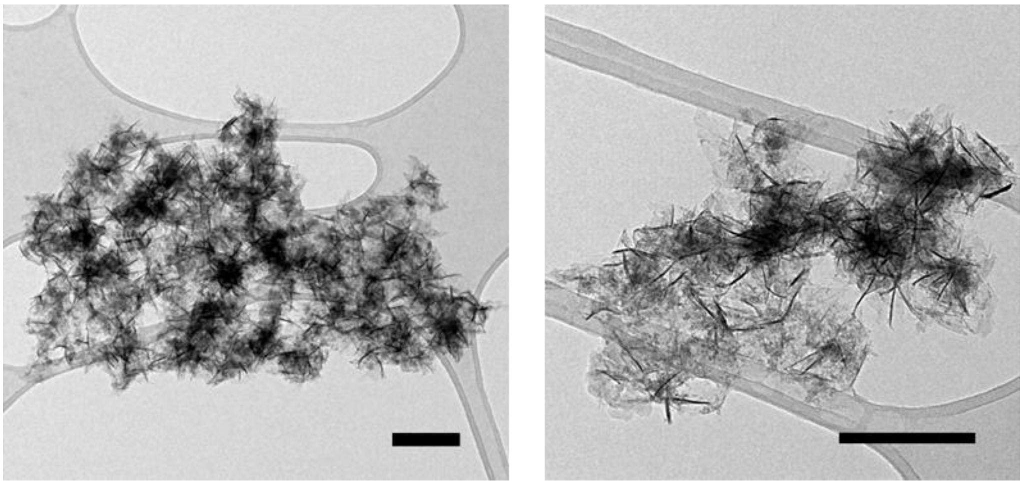

Figure S17. TEM images of FeOOH@Tz-COF. Scale bar equals 200 nm.

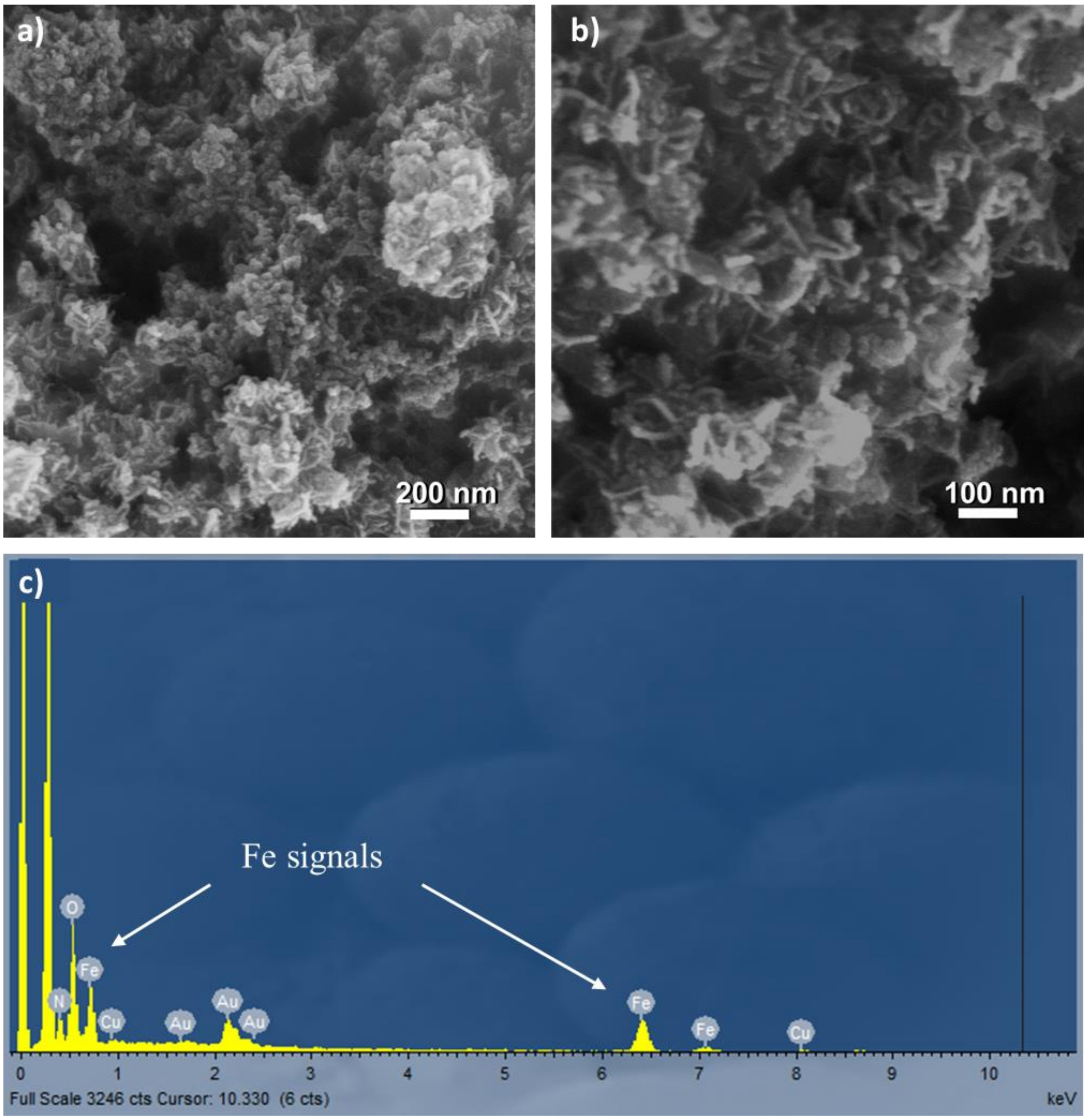

Figure S18. SEM images of FeOOH@Tz-COF (a-b) and EDX spectrum of the material (c)

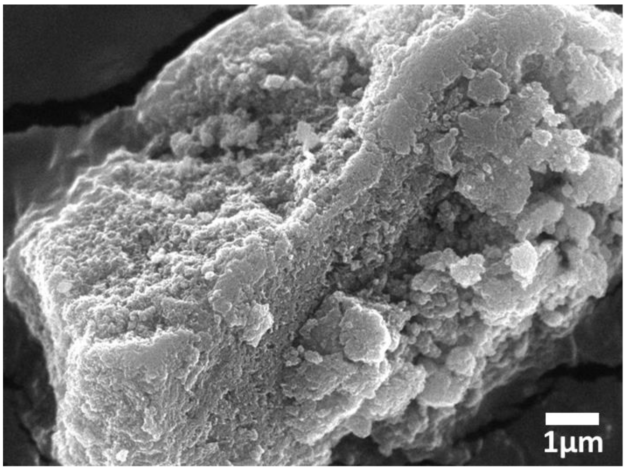

Figure S19. SEM image of FeOOH synthesized without Tz-COF nanoparticles.

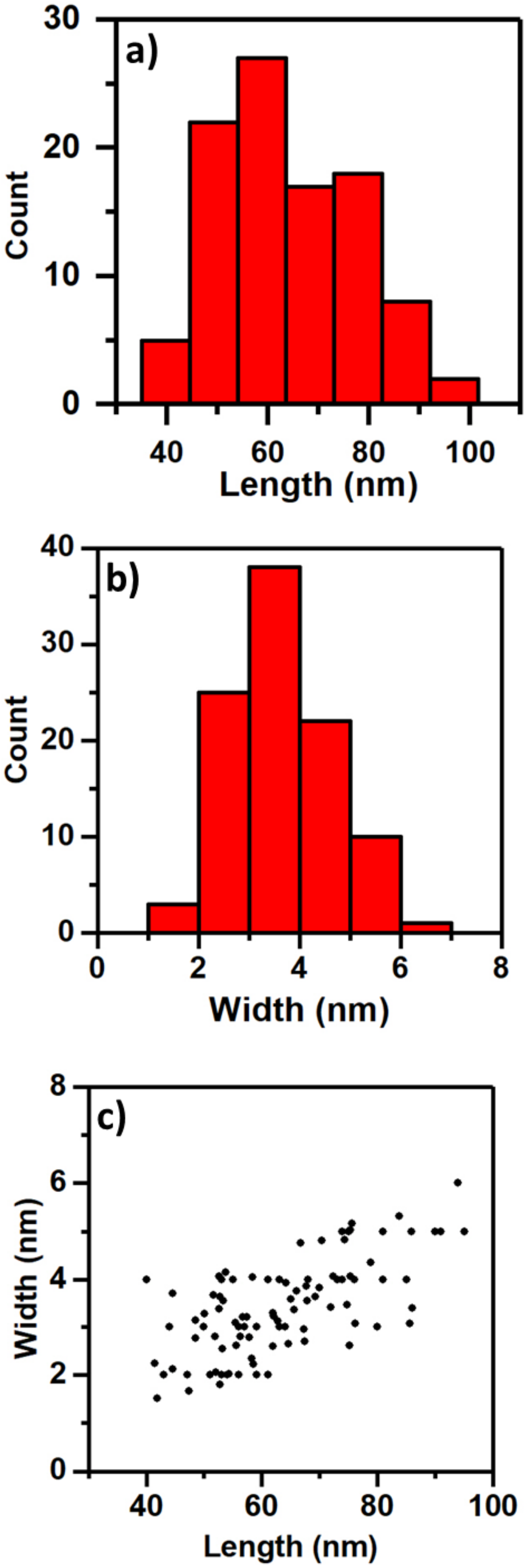

Figure S20. Statistical study of the size of lepidocrocite nanorods in FeOOH@Tz-COF.

- **Characterization of Tz-COF(s) and FeOOH@Tz-COF by XPS.**

The complete XPS spectra of the two samples have been taken and analyzed to check the expected composition. Fig. S21 shows the XPS overview spectra for Tz-COF(s) and FeOOH@Tz-COF with the most important peaks labelled. No additional peaks from contamination are observed. The ratio between the different species agrees with the compound compositions weighted by the cross-sections of each peak.

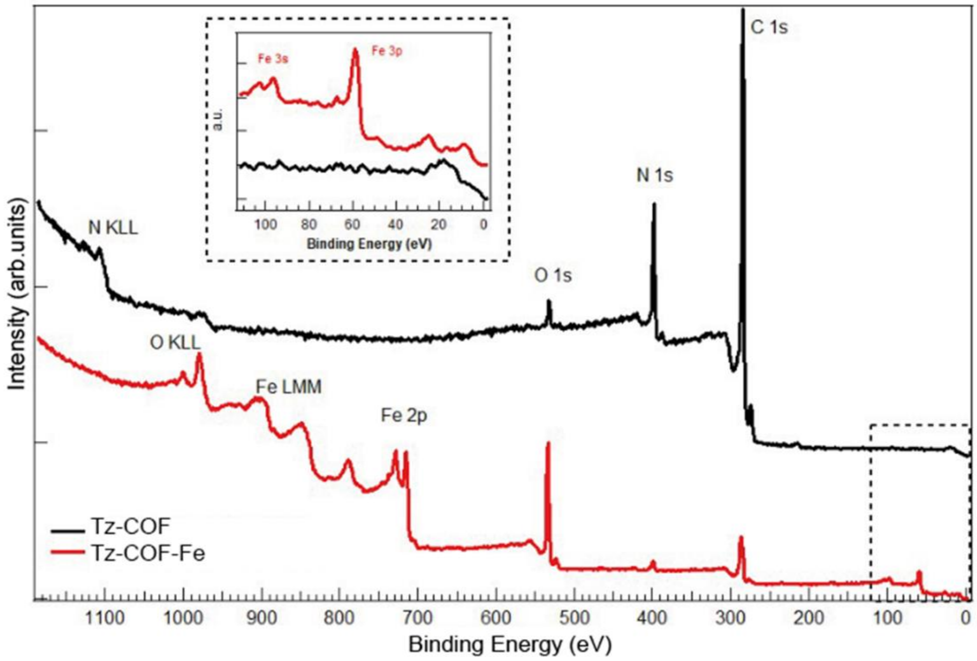

Figure S21. XPS spectra of Tz-COF(s) (black) and FeOOH@Tz-COF (red). The most important core levels and Auger peaks are labelled. The low energy region (with Fe 3s and Fe 3p peaks) is enlarged in the inset, due to its low relative intensity compared to the rest of the spectrum. Tz-COF(s) spectrum is vertically displaced to avoid superposition in the main panel (inset). Note the appearance of Fe peaks in the FeOOH@Tz-COF sample.

The detailed spectra of the O 1s and Fe 2p core levels are crucial for the determination of the Fe oxide species[2] formed upon reaction, as during the synthesis of the composite different iron oxide species such as hematite (α-Fe_2O_3), maghemite (γ-Fe_2O_3), goethite (α-FeOOH), lepidocrocite (γ-FeOOH) or magnetite (Fe_3O_4) may have been formed. The most obvious difference in their relevant XPS core level positions for these compounds is the O 1s components in the oxide-hydroxide compounds (goethite and lepidocrocite).

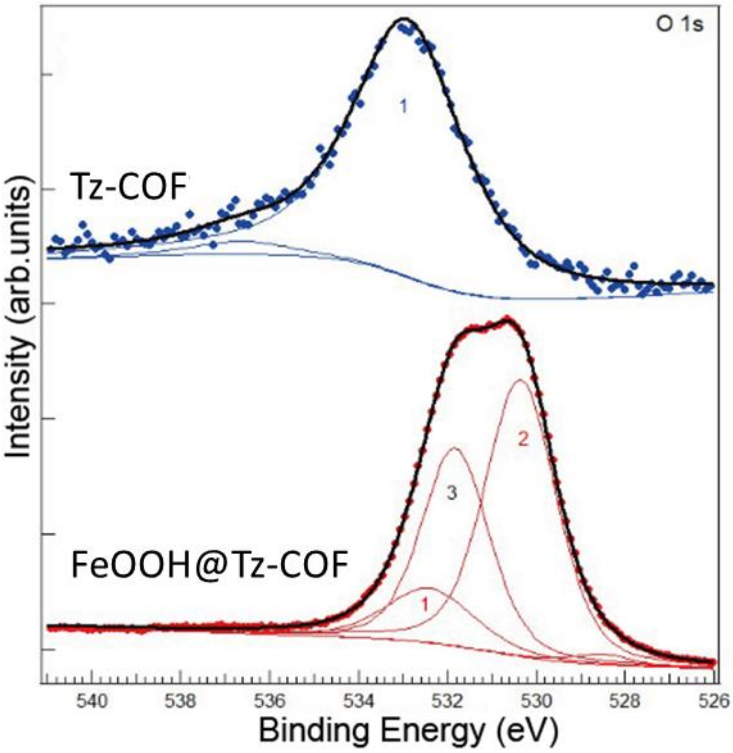

Figure S22. XPS O 1s core-level spectra of Tz-COF(s) and FeOOH@Tz-COF, including a line shape analysis and deconvolution of the peaks. Note that the O 1s intensity of Tz-COF(s) is multiplied by 15, compared to FeOOH@Tz-COF.

Fig S22 shows the O 1s spectra of the two samples. The Tz-COF sample has a low oxygen contribution and an almost single component O 1s. In turn, the FeOOH@Tz-COF sample presents an intense O 1s core level with two distinct main components. This observation discards magnetite, hematite, and maghemite oxides as possible Fe oxides formed during the reaction because in these oxides, there is a single O 1s component.[2] O 1s peak has been fitted to verify this hypothesis (Fig. S22). Note that in the original data, the intensity for O 1s in Tz-COF(s) is much lower and has a lower resolution, resulting in a worse fit. This explains the increased width of the single component seen in this peak. This component can be attributed to residual C=O bonds from the reactants. The O 1s signal from FeOOH@Tz-COF corresponds to the two oxygen components expected for the Fe oxide: a component at 530.1 eV corresponding to the O^{2-} and a component at 531.9 eV that corresponds to the OH^{-}.

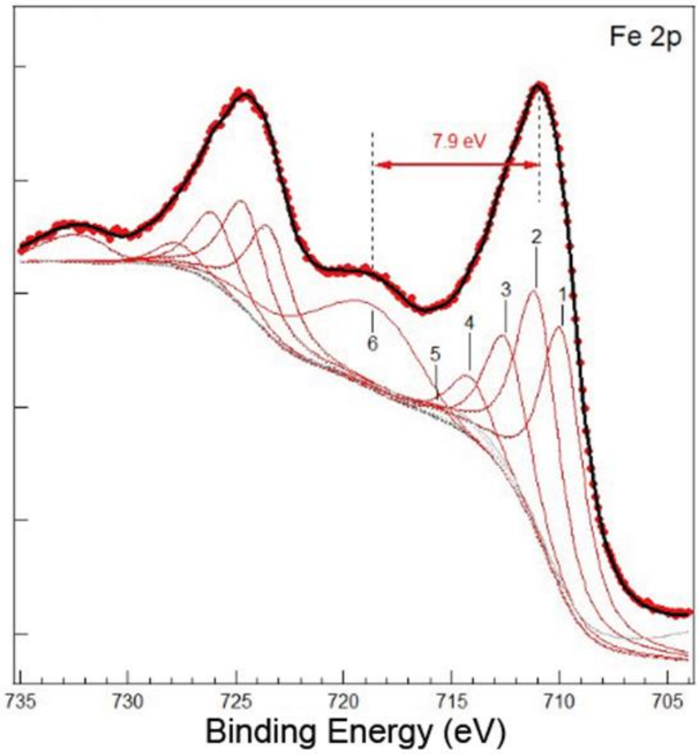

Figure S23. XPS data for Fe 2p core level of the modified FeOOH@Tz-COF. The deconvolution is based on Ref. 2. The main peak - satellite distance (red double arrow) is taken from the visual maxima in each case for comparison with other reported data.

Now the problem remains to discern between goethite and lepidocrocite. For this, we can rely on the Fe 2p core level, shown in Fig. S23, including a line shape analysis made according to literature. The most significant difference between the remaining Fe compounds is the distance to the satellite peak. This value amounts to 8.0 eV for lepidocrocite and 8.4 eV for goethite. Given the value found (7.9 eV), and the good fit obtained using the lepidocrocite components, we conclude that the Fe compound present in the sample is γ- FeOOH (lepidocrocite) with a very high degree of certitude.

Section S3. Elimination of As(III) in water with FeOOH@Tz-COF nanocomposite

- **As(III) uptake capacity of FeOOH@Tz-COF. Freundlich isotherm**

Table S3. As(III) adsorption data for FeOOH@Tz-COF.V=15 mL, contact time: 3 h, mCOF=1.5 mg, T=25 ºC.

$[As]_{initial}$ (ppm)	$[As]_{final}$ (ppm)	Q_e (mg ads/g COF)
0.5	0.0802	4.20
3	0.898	21.02
6	2.705	32.95
15	9.248	57.52
30	21.327	87.73
90	68.606	213.94
120	98.822	271.78

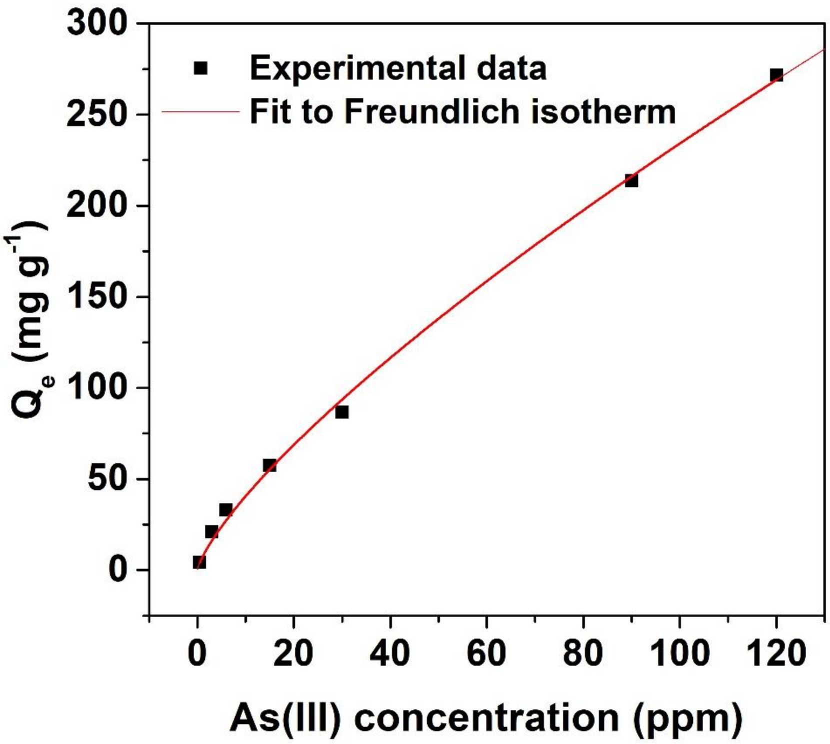

Figure S24. Freundlich plot for the adsorption of As(III) in FeOOH@Tz-COF.

Kinetic studies

Table S4. Kinetics investigation data for FeOOH@Tz-COF As(III) adsorption. As(III) initial concentration of 1.75 mg L^{-1}, V = 15 mL, mCOF = 1.5 mg, T = 25 ºC.

time (min)	$[As]_{final}$ (ppm)	q_t (mg ads/g COF)	t/q_t (min mg ads/g COF)
0	1.75	0.00	0.00
5	0.733	10.17	0.492
10	0.705	10.45	0.957
20	0.688	10.62	1.883
30	0.68	10.70	2.804
60	0.672	10.78	5.566
180	0.618	11.32	15.901

Table S5. Comparison of different reported adsorbents used for the removal of arsenic from water

Adsorbents	Q_e (mg/g)	k_2 (g mg^{-1} min^{-1})	ref
MgO-nflakes	506.6	0.0012	3
ZVI-MNCs	47.45	0.001	4
ZVIM	58.90	0.043	5
HP-UiO-66	248.75	0.0002	6
UiO-66-36TFA	200	0.0015	7
CINs	267.2	0.003	8
MBOP	16.94	0.0049	9
β-FeOOH/CF	103.4	0.0019	10
Fe-Cu binary oxide	122.3	0.0016	11
UiO-66-SH-A	90.7	0.013	12
GN-α-FeOOH aerogel	13.42	0.011	13
FeMnOx/RGO	47.05	0.0075	14
FeOOH@Tz-COF	272	0.058	This work

- **pH studies**

Table S6. As(III) uptake data at different pH for FeOOH@Tz-COF. As(III) initial concentration of 1.75 mg L^{-1}, V = 25 mL, mCOF = 5 mg, T = 25ºC, contact time = 3 h.

pH	$[As]_{final}$ (ppm)	Q_e (mg ads/g COF)
3	0.99	3.78
5	0.37	6.88
7	0.27	7.41
9	0.33	7.13
11	0.47	6.42

- **Interference test**

Table S7. Adsorption selectivity test of FeOOH@Tz-COF towards As(III) in the presence of different cations and anions. The conditions for each experiment are: V = 25 mL, mCOF = 5 mg, T = 25 ºC, contact time = 3 h.

	Ions	$[C]_{initial}$ (ppm)	$[C]_{final}$ (ppm)	% Adsorbed
**Cation study**	**As(III)**	**1.40**	**0.26**	**81.21**
	Cd(II)	1.53	1.45	5.29
	Pb(II)	1.48	0.028	98.11
	Hg(II)	1.05	0.178	82.9
	Mg(II)	1.62	1.70	0
	Zn(II)	1.59	1.60	0
	Na(I)	8.39	8.5	0
**Anion study**	**As(III)**	**1.39**	**0.463**	**66.69**
	$H_2PO_4^-$	1.77	0.063	96.44
	Cl^-	0.97	0.99	0
	SiO_3^{2-}	0.3	0.35	0

- **Other contaminants: Pb(II) and Hg(II) uptake capacity of FeOOH@Tz-COF**

Table S8. Pb(II) adsorption data for FeOOH@Tz-COF. V=15 mL, contact time =3 h, mCOF = 1.5 mg, T = 25 ºC.

$[Pb]_{initial}$ (ppm)	$[Pb]_{final}$ (ppm)	Q_e (mg ads/g COF)
0.432	0.000024	4.320
2.594	0.046	25.48
5.187	0.827	43.60
12.96	8.525	44.44
25.94	18.55	73.92
43.23	36.89	63.40

Table S9. Hg(II) adsorption data for FeOOH@Tz-COF. V = 15 mL, contact time = 3 h, mCOF = 1.5 mg, T = 25 ºC.

$[Hg]_{initial}$ (ppm)	$[Hg]_{final}$ (ppm)	Q_e (mg ads/g COF)
0.694	0.029	6.65
4.164	0.763	34.01
20.820	14.58	62.38
40.12	28.20	119.2
69.4	50.96	184.4

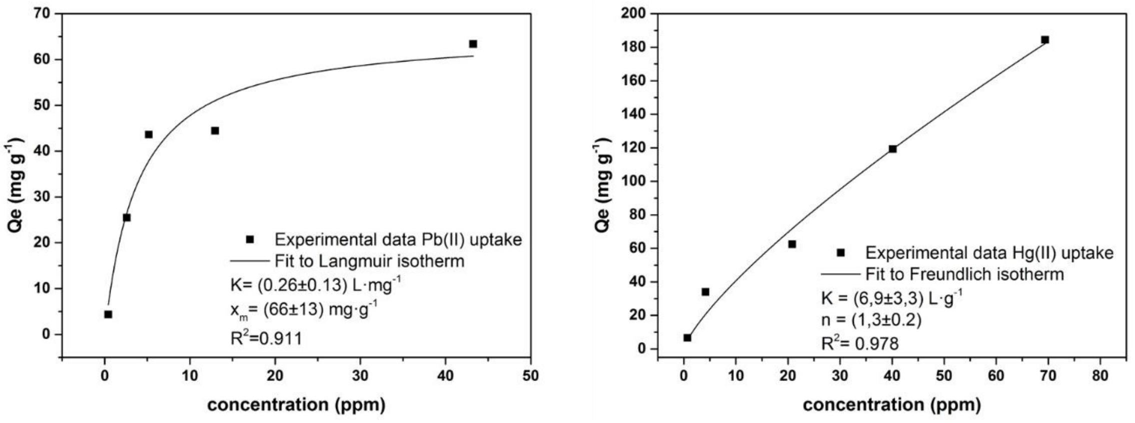

Figure S25. Langmuir plot for the adsorption of Pb(II) in FeOOH@Tz-COF (left) and Freundlich plot for the adsorption of Hg(II) in FeOOH@Tz-COF (right).

- **Regeneration studies**

Table S10. Recyclability data of FeOOH@Tz-COF. As(III) initial concentration of 1.75 mg L^{-1}, V = 25 mL, mCOF = 15 mg, T = 25 ºC, contact time = 3 h.

Cycle	% Regeneration
I	100
II	98.9
III	104.4
IV	93.4

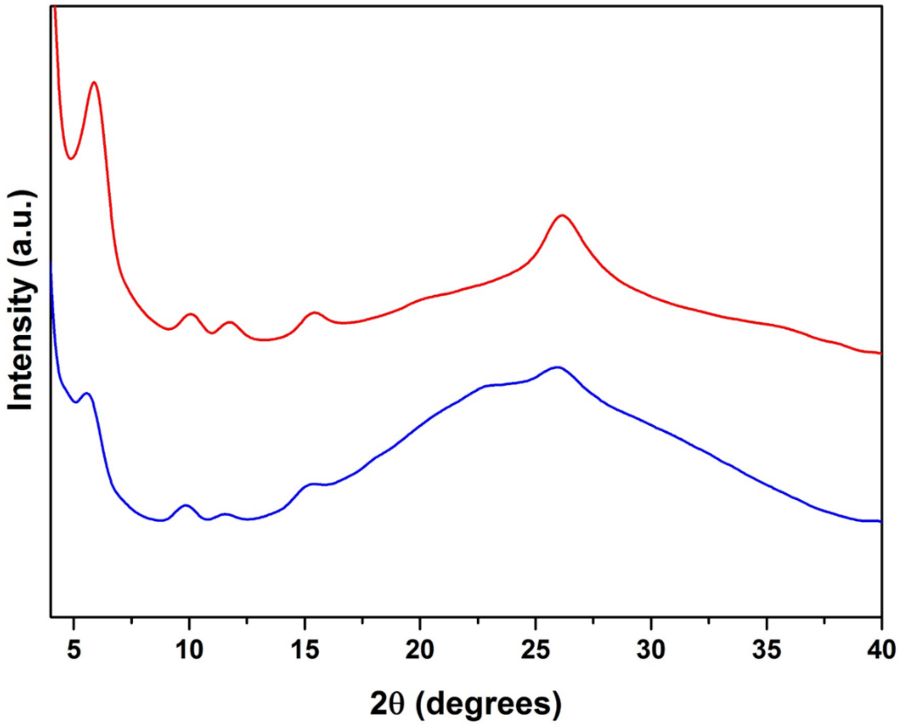

Figure S26. PXRD patterns of FeOOH@Tz-COF before the As(III) adsorption (red) and FeOOH@Tz-COF after the forth recycling cycle.

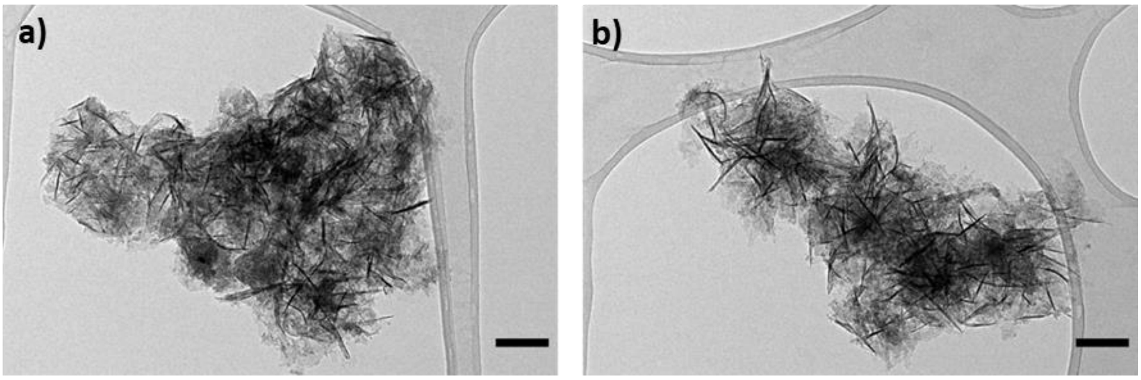

Figure S27. TEM images of FeOOH@Tz-COF after four recycling cycles. Scale bar equals 200 nm.

References

(1) Schmid, M.; Steinrück, H.-P.; Gottfried, J. M. A New Asymmetric Pseudo-Voigt Function for More Efficient Fitting of XPS Lines. *Surf. Interface Anal.* **2014**, *46* (8), 505–511. https://doi.org/10.1002/sia.5521.

(2) Grosvenor, A. P.; Kobe, B. A.; Biesinger, M. C.; McIntyre, N. S. Investigation of Multiplet Splitting of Fe 2p XPS Spectra and Bonding in Iron Compounds. *Surf. Interface Anal.* **2004**, *36* (12), 1564–1574. https://doi.org/10.1002/sia.1984.

(3) Liu, Y.; Li, Q.; Gao, S.; Shang, J. K. Exceptional As(III) Sorption Capacity by Highly Porous Magnesium Oxide Nanoflakes Made from Hydrothermal Synthesis. *J. Am. Ceram. Soc.* **2011**, *94* (1), 217–223. https://doi.org/10.1111/j.1551-2916.2010.04043.x.

(4) Zubair, Y. O.; Fuchida, S.; Tokoro, C. Insight into the Mechanism of Arsenic(III/V) Uptake on Mesoporous Zerovalent Iron–Magnetite Nanocomposites: Adsorption and Microscopic Studies. *ACS Appl. Mater. Interfaces* **2020**, *12* (44), 49755–49767. https://doi.org/10.1021/acsami.0c14088.

(5) Panda, A. P.; Rout, P.; Kumar, S. A.; Jha, U.; Swain, S. K. Enhanced Performance of a Core–Shell Structured Fe(0)@Fe Oxide and Mn(0)@Mn Oxide (ZVIM) Nanocomposite towards Remediation of Arsenic Contaminated Drinking Water. *J. Mater. Chem. A* **2020**, *8* (8), 4318–4333. https://doi.org/10.1039/D0TA00611D.

(6) Xu, R.; Ji, Q.; Zhao, P.; Jian, M.; Xiang, C.; Hu, C.; Zhang, G.; Tang, C.; Liu, R.; Zhang, X.; Qu, J. Hierarchically Porous UiO-66 with Tunable Mesopores and Oxygen Vacancies for Enhanced Arsenic Removal. *J. Mater. Chem. A* **2020**, *8* (16), 7870–7879. https://doi.org/10.1039/C9TA13747E.

(7) Assaad, N.; Sabeh, G.; Hmadeh, M. Defect Control in Zr-Based Metal-Organic Framework Nanoparticles for Arsenic Removal from Water. *ACS Appl. Nano Mater.* **2020**, *3* (9), 8997–9008. https://doi.org/10.1021/acsanm.0c01696.

(8) Gerard, N.; Santhana Krishnan, R.; Ponnusamy, S. K.; Cabana, H.; Vaidyanathan, V. K. Adsorptive Potential of Dispersible Chitosan Coated Iron-Oxide Nanocomposites

toward the Elimination of Arsenic from Aqueous Solution. *Process Saf. Environ. Prot.* **2016**, *104*, 185–195. https://doi.org/10.1016/j.psep.2016.09.006.

(9) Dhoble, R. M.; Lunge, S.; Bhole, A. G.; Rayalu, S. Magnetic Binary Oxide Particles (MBOP): A Promising Adsorbent for Removal of As (III) in Water. *Water Res.* **2011**, *45* (16), 4769–4781. https://doi.org/10.1016/j.watres.2011.06.016.

(10) Ge, X.; Ma, Y.; Song, X.; Wang, G.; Zhang, H.; Zhang, Y.; Zhao, H. β-FeOOH Nanorods/Carbon Foam-Based Hierarchically Porous Monolith for Highly Effective Arsenic Removal. *ACS Appl. Mater. Interfaces* **2017**, *9* (15), 13480–13490. https://doi.org/10.1021/acsami.7b01275.

(11) Zhang, G.; Ren, Z.; Zhang, X.; Chen, J. Nanostructured Iron(III)-Copper(II) Binary Oxide: A Novel Adsorbent for Enhanced Arsenic Removal from Aqueous Solutions. *Nanotechnol. Water Wastewater Treat.* **2013**, *47* (12), 4022–4031. https://doi.org/10.1016/j.watres.2012.11.059.

(12) Shao, P.; Ding, L.; Luo, J.; Luo, Y.; You, D.; Zhang, Q.; Luo, X. Lattice-Defect-Enhanced Adsorption of Arsenic on Zirconia Nanospheres: A Combined Experimental and Theoretical Study. *ACS Appl. Mater. Interfaces* **2019**, *11* (33), 29736–29745. https://doi.org/10.1021/acsami.9b06041.

(13) Andjelkovic, I.; Tran, D. N. H.; Kabiri, S.; Azari, S.; Markovic, M.; Losic, D. Graphene Aerogels Decorated with α-FeOOH Nanoparticles for Efficient Adsorption of Arsenic from Contaminated Waters. *ACS Appl. Mater. Interfaces* **2015**, *7* (18), 9758–9766. https://doi.org/10.1021/acsami.5b01624.

(14) Zhu, J.; Lou, Z.; Liu, Y.; Fu, R.; Baig, S. A.; Xu, X. Adsorption Behavior and Removal Mechanism of Arsenic on Graphene Modified by Iron–Manganese Binary Oxide (FeMnOx/RGO) from Aqueous Solutions. *RSC Adv.* **2015**, *5* (83), 67951–67961. https://doi.org/10.1039/C5RA11601E.
